# Genomic Epidemiology of Hypervirulent Serogroup W, ST-11 *Neisseria meningitidis*

**DOI:** 10.1016/j.ebiom.2015.09.007

**Published:** 2015-09-08

**Authors:** Mustapha M. Mustapha, Jane W. Marsh, Mary G. Krauland, Jorge O. Fernandez, Ana Paula S. de Lemos, Julie C. Dunning Hotopp, Xin Wang, Leonard W. Mayer, Jeffrey G. Lawrence, N. Luisa Hiller, Lee H. Harrison

**Affiliations:** a Infectious Diseases Epidemiology Research Unit, University of Pittsburgh, Pittsburgh, PA 15261, USA; b Molecular Genetics Laboratory, Public Health Institute of Chile, Santiago, Chile; c Department of Bacteriology, Instituto Adolfo Lutz (IAL), São Paulo, Brazil; d The Institute for Genome Sciences, University of Maryland School of Medicine, University of Maryland, Baltimore, MD, USA; e Meningitis and Vaccine Preventable Diseases Branch, Division of Bacterial Diseases, National Center for Immunization and Respiratory Diseases, Centers for Disease Control and Prevention, Atlanta, GA, USA; f Department of Biological Sciences, University of Pittsburgh, USA; g Department of Biological Sciences, Carnegie Mellon University, Pittsburgh, PA, USA; h Public Health Dynamics Laboratory, Graduate School of Public Health, University of Pittsburgh, USA

**Keywords:** Hajj clone, Invasive meningococcal disease, W135, Whole genome sequencing, Virulence factors, FHbp

## Abstract

*Neisseria meningitidis* is a leading bacterial cause of sepsis and meningitis globally with dynamic strain distribution over time. Beginning with an epidemic among Hajj pilgrims in 2000, serogroup W (W) sequence type (ST) 11 emerged as a leading cause of epidemic meningitis in the African ‘meningitis belt’ and endemic cases in South America, Europe, Middle East and China. Previous genotyping studies were unable to reliably discriminate sporadic W ST-11 strains in circulation since 1970 from the Hajj outbreak strain (Hajj clone). It is also unclear what proportion of more recent W ST-11 disease clusters are caused by direct descendants of the Hajj clone. Whole genome sequences of 270 meningococcal strains isolated from patients with invasive meningococcal disease globally from 1970 to 2013 were compared using whole genome phylogenetic and major antigen-encoding gene sequence analyses. We found that all W ST-11 strains were descendants of an ancestral strain that had undergone unique capsular switching events. The Hajj clone and its descendants were distinct from other W ST-11 strains in that they shared a common antigen gene profile and had undergone recombination involving virulence genes encoding factor H binding protein, nitric oxide reductase, and nitrite reductase. These data demonstrate that recent acquisition of a distinct antigen-encoding gene profile and variations in meningococcal virulence genes was associated with the emergence of the Hajj clone. Importantly, W ST-11 strains unrelated to the Hajj outbreak contribute a significant proportion of W ST-11 cases globally. This study helps illuminate genomic factors associated with meningococcal strain emergence and evolution.

## Highlights

• Genomic characterization of serogroup W ST-11 of *Neisseria meningitidis.*
.
• Epidemic W ST-11 strain (Hajj clone) emerged through recombination affecting virulence genes.
• Both the Hajj clone and W ST-11 strains unrelated to the Hajj outbreak have persisted globally.

Neisseria meningitidis, a bacterial cause of frequently fatal brain (meningitis) and blood stream (sepsis) infections, has variable strain distribution over time. Serogroup W sequence type 11 (W ST-11) lineage is associated on one hand with strains causing only rare (sporadic) disease cases, and the Hajj clone – a major global cause of epidemic and endemic meningococcal disease. In this study we analyzed complete genome sequences of a global collection of 270 W ST-11 isolates causing meningococcal disease from 1970-2013. The Hajj clone acquired novel gene sequences within genes involved in nitrogen metabolism (nitrogen oxide reductase, nitrite reductase) and evasion of human immune response (factor H binding protein). These genes may be the cause of increased virulence of the Hajj clone and can be used to trace continuing spread of the clone. These results shed light on mechanisms of meningococcal strain emergence.

## 1 Introduction

*Neisseria meningitidis* is a major global cause of meningitis and sepsis with large variations in disease incidence rates and strain distribution globally (Halperin et al., 2012). Incidence rates range from 0.5–15 cases per 100,000 population across most global regions. Very high incidence rates of 100–1000 per 100,000 are witnessed during occasional epidemics across 21 countries (Multi-Disease Surveillance Centre Ouagadougou RMS, 2002–2015) in Africa collectively referred to as the ‘meningitis belt’(Lapeyssonnie, 1968). Meningococci are classified into serogroups based on biochemical properties of their polysaccharide capsule — the primary determinant of meningococcal virulence and major vaccine target. Serogroups A, B, C, W (formerly W-135) and Y cause almost all invasive disease cases. Other virulence determinants are lipooligosaccharide and several outer membrane proteins (Stephens, 2009). Multilocus sequence typing (MLST) (Maiden et al., 1998), based on DNA sequence of seven housekeeping genes, is used to classify meningococci into lineages (sequence types, ST). Closely related STs are termed ‘clonal complex.’

Outer membrane proteins, porins A and B (PorA, PorB) (Russell et al., 2004, Tanabe et al., 2010) and iron-regulated enterobactin (FetA) (Thompson et al., 2003) are used for “fine typing” of meningococcal isolates while factor H binding protein (FHbp) (Seib et al., 2015), *Neisserial* adhesion A (NadA) (Capecchi et al., 2005) and *Neisserial* heparin binding antigen (Nhba) (Serruto et al., 2010) are components of capsule-independent vaccines developed for prevention of serogroup B disease (Granoff, 2010). Meningococci have a very plastic genome as a result of frequent acquisition of genetic material from other *Neisseria* or more distant bacterial species through recombination (Kong et al., 2013; Holmes et al., 1999). Recombination involving major outer membrane antigen genes — “antigenic shift” has been linked to increased incidence of meningococcal disease (Harrison et al., 2006). Capsular switching – acquisition of novel capsule specific genes through recombination has also facilitates the emergence and persistence of virulent meningococcal lineages (Swartley et al., 1997, Harrison et al., 2010). Through capsular switching, defined as presence of different capsular phenotypes within the same clonal complex, meningococcal strains belonging to virulent lineages may escape vaccine induced immunity.

In the 1970s to 1990s, serogroup W was a rare cause of meningococcal disease. In 2000, the first recorded serogroup W meningococcal disease outbreak occurred during the annual Hajj pilgrimage in Mecca, Saudi Arabia (Taha et al., 2000, Aguilera et al., 2002). The Hajj outbreak strain, referred to as the Hajj clone, was characterized as belonging to the hypervirulent sequence type (ST)-11 genetic lineage and having the PorA antigen-encoding gene type P1.5,2 (Mayer et al., 2002). Since 2000, W ST-11 strains that are genetically similar to the Hajj clone have caused large epidemics in the African meningitis belt (Decosas and Koama, 2002, Collard et al., 2010, MacNeil et al., 2014) and have caused case clusters and smaller outbreaks in South Africa (von Gottberg et al., 2008), China (Zhou et al., 2013), Taiwan (Chiou et al., 2006), Brazil (Lemos et al., 2010), and most recently in Argentina (Efron et al., 2009), Chile (Barra et al., 2013) and the United Kingdom (Ladhani et al., 2015).

A majority of ST-11 strains isolated in 1960–1999 expressed serogroup C capsule (http://pubmlst.org/neisseria/). As a result, W ST-11 is thought to have emerged from serogroup C ST-11 lineage through capsular switching though the direction of capsular switching has not been established (Kelly and Pollard, 2003). The Hajj clone was highly similar by most molecular typing techniques including pulsed field gel electrophoresis (PFGE), PorA and FetA genotyping to historical rare sporadic group W ST-11 strains isolated globally from 1970 to 1999 (Mayer et al., 2002, Fonkoua et al., 2002, Taha et al., 2004). These “pre-Hajj” strains were distinguished by 16S ribosomal RNA (rRNA) genotyping as 16S type 13 and type 14 compared to type 31 associated with the Hajj clone (Mayer et al., 2002). The high genetic and antigenic similarity between the Hajj clone and pre-Hajj W ST-11 strains by traditional molecular techniques markedly contrasts with the abrupt and dramatic changes in the epidemiology of W ST-11 that began with the Hajj outbreak in 2000 (Kelly and Pollard, 2003).

In a recent study, we compared capsular gene sequences from the Hajj clone and 24 other invasive W ST-11 strains isolated from 1970 to 2012 (Mustapha et al., 2014). Phylogenetic and BLAST analyses against a database of over 2300 genome sequences demonstrated that the Hajj clone and 24 other invasive W ST-11 strains shared identical capsular recombination events, with a sporadic group W strain and Y ST-23 as the most likely donor lineages into a serogroup C ST-11 strain (Mustapha et al., 2014). Taken together, these studies suggest that historical sporadic W ST-11 strains and the Hajj clone have a common origin and likely emerged from a capsular switching event in a single ancestral C ST-11 strain. Furthermore, these studies suggested that genetic differences outside the capsule region may be responsible for increased virulence seen in the Hajj clone when compared to historical sporadic W ST-11 strains.

Whole genome sequencing (WGS) could potentially illuminate genetic differences not detectable by older genotyping techniques that could account for the observed epidemiologic differences between W ST-11 strains. We analyzed whole genome sequencing (WGS) data from invasive serogroup W ST-11 strains isolated globally from 1970 to 2013 to generate a model of W ST-11 evolution and global spread. We postulated that subtle genetic variations through recombination and/or mutation, outside of the capsule gene cluster may distinguish these W ST-11 strains and could explain the dramatic emergence and increased virulence of the Hajj clone. We identified genomic markers that are unique to the Hajj clone, traced continued global spread of the Hajj clone, and interestingly, found that some of the current W ST-11 case clusters are caused by W ST-11 strains that are not direct descendants of the Hajj clone.

## 2 Methods

### 2.1 Strain selection

We analyzed 270 *N.meningitidis* serogroup W ST-11 genomes isolated from disease cases over four decades (1970–2013). Twenty-six genomes were newly sequenced while 244 genome sequences were identified from public databases (Supplementary Tables 1 and 2). All 26 newly sequenced genomes including 14 genomes that were part of the Genomic Sequencing Center for Infectious Disease (GSCID, http://gscid.igs.umaryland.edu/) project are made publically available in both PubMLST (http://pubmlst.org/neisseria/) and GenBank databases. In the PubMLST database, 273 genome sequences were designated serogroup W ST-11 by the end of June 2014, of which 31 genomes were excluded because they had missing information in at least one of six antigen gene alleles. Two genomes were obtained from *Neisseria* Base (http://nbase.biology.gatech.edu/) (Katz et al., 2011, Jolley and Maiden, 2010). Geographically, a majority of strains were isolated in the United Kingdom (52.2%, n = 141), South Africa (36.3%, n = 98), the ‘meningitis belt’ (4.1%, n = 11) or South America (2.6%, n = 7).

### 2.2 Strain classification

**Fig. 1:**
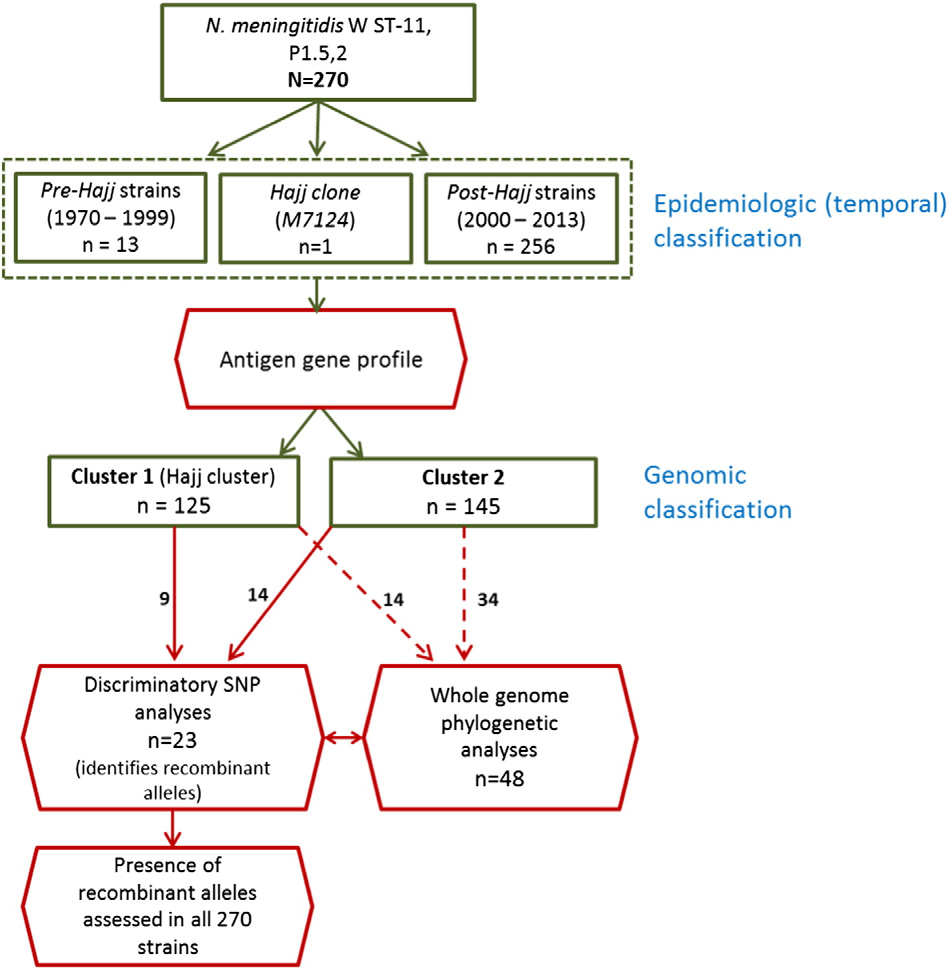
Flow chart showing how 270 invasive W ST-11 meningococcal strains were grouped based on date of isolation (epidemiologic classification) and the combined results of whole genome sequence analysis (genomic classification). All 270 strains were classified into Cluster 1 and 2 based on antigen-encoding gene analyses and presence of recombinant genomic regions. Several strains from each cluster were further analyzed by whole genome phylogenetic and Hajj specific SNP analyses as described in the Methods.

W ST-11 invasive meningococcal strains were classified as isolated before, during or after the Hajj 2000 outbreak (Fig. 1). Pre-Hajj strains (n = 13) were isolated globally from 1970 to 1999, the Hajj clone (n = 1) (Strain ID: *M7124*) was isolated in Saudi Arabia during the Hajj outbreak in 2000 (Mayer et al., 2002), post-Hajj (n = 256) were isolated during 2000–2013. All 270 whole genome sequences were characterized using antigen-encoding gene profiles and presence of genomic regions associated with allelic exchange. Forty-eight isolates representative of the antigen-encoding gene and geographic diversity of all 270 strains were selected for whole genome phylogenetic analyses.

### 2.3 Whole genome sequencing, assembly and genome annotation

A total of 26 W ST-11 isolates were newly sequenced using Pacific Biosciences RS II (PacBio, www.pacificbiosciences.com) Single Molecule Real Time (SMRT) sequencing (n = 7), paired-end Illumina HiSeq only (Illumina, www.illumina.com, n = 11), paired-end Illumina and Roche 454 (http://454.com/) pyrosequencing (Illumina + 454, n = 3) and Life Technologies (www.lifetechnologies.com) Ion Torrent PGM sequencing (n = 6) platforms (Supplementary Table 1). Library preparation and sequencing were done according to manufacturer protocols. *M7124* was re-sequenced using PacBio to provide very high quality Hajj reference genome sequence.

*De novo* assembly was done using Hierarchical Genome Assembly Process (HGAP) v4.0 for PacBio, Celera v7.0 for Illumina/454 and Mira 3.0 for Ion Torrent with default settings and assembly qualities were checked as described in Supplementary Methods (Chin et al., 2013, Chevreux et al., 2004). Contiguous genomic DNA sequences (contigs) from *de novo* assemblies were annotated on RAST and IGS annotation servers (Aziz et al., 2008). Assembled contig files were downloaded from public databases for the remaining 244 genome sequences (Supplementary Table 2).

### 2.4 Whole genome alignment and phylogenetic analyses

Forty-eight representative W ST-11 strains underwent whole genome phylogenetic analyses. Core genome alignment of 1,014,185 sites was generated using Mauve v2.3 (Darling et al., 2010) and a Maximum-Likelihood phylogenetic tree was constructed using general time reversible model with invariant sites (GTR + Γ + I) with 100 bootstrap replicates using PhyML v3.0 (Guindon et al., 2010). The effect of recombination on phylogenetic relationships was assessed using SplitsTree v4 (Huson and Bryant, 2006) and ClonalFrame v1.2 (Vos and Didelot, 2009). ClonalFrame is a phylogenetic reconstruction method that determines phylogenetic relationships between bacterial strains after detecting and accounting for recombinant sequences based on a coalescent model of evolution. SplitsTree v4 uses the neighbor-net algorithm that determines phylogenetic networks using an agglomerative method (Huson and Bryant, 2006, Bryant and Moulton, 2004). Unweighted mean pairwise distances were obtained from SplitsTree v4.

**Fig. 2:**
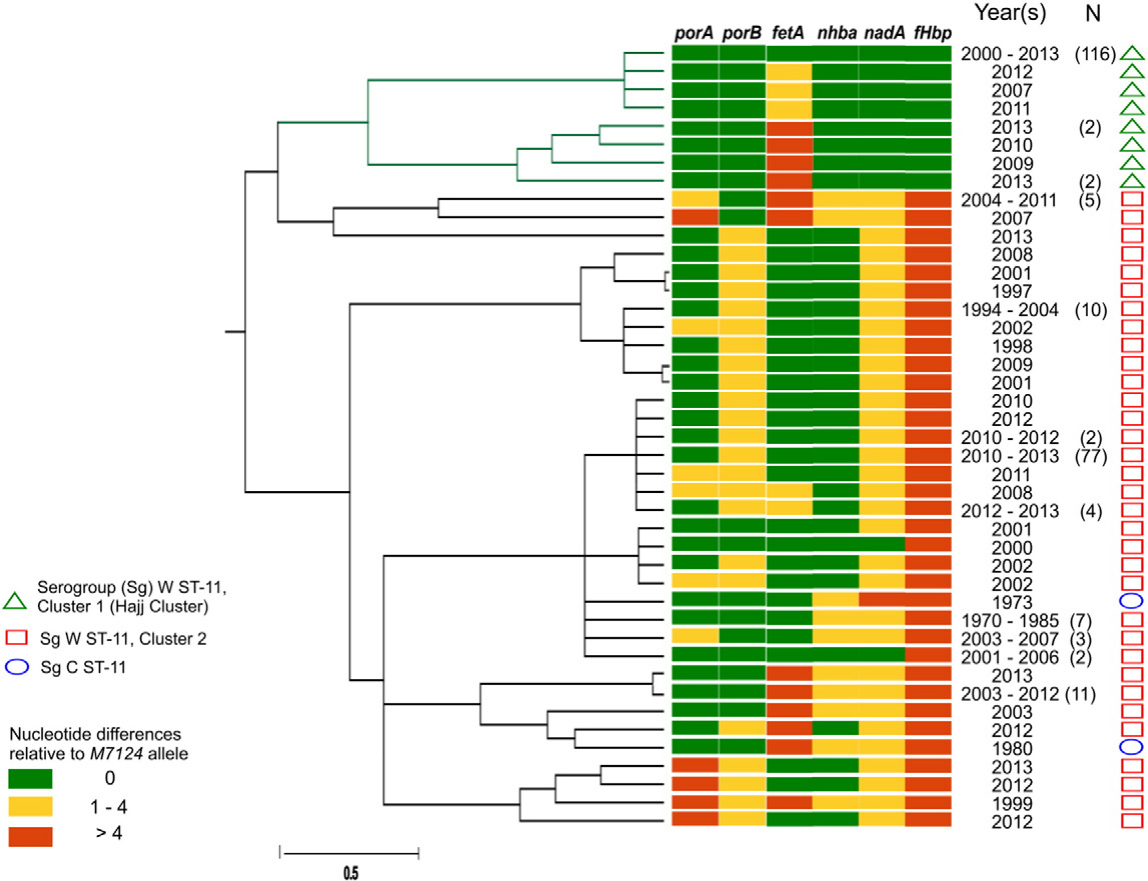
Antigen-encoding gene profiles of 270 invasive serogroup W and historical serogroup C ST-11 strains. On the left is ClonalFrame consensus tree constructed using concatenated full length antigen-encoding gene sequences from *porA*, *porB*, *fetA*, *nadA*, *nhba* and *fHbp* genes. Color chart in the center depicts antigen gene allelic differences among W ST-11 strains compared to *M7124* allele. Year(s) reflect earliest and most recent isolation dates for strains with the listed antigen-encoding gene profile; Numbers in parentheses indicate total number of strains with identical antigen-encoding gene profile to the adjacent strain for profiles shared by more than one strain. On the right, green open triangles mark Cluster 1 strains, red open squares mark Cluster 2 and blue open circles mark serogroup C ST-11 strains. Antigen-encoding gene allele numbers were obtained from www.pubmlst.org/neisseria. Scale bar represents time (coalescent units).

Antigen gene allele designations for full length *porA*, *porB*, *fetA*, *nadA*, *nhbA* and *fHbp* genes were obtained by comparing assembled contigs to reference alleles downloaded from the *Neisseria* PubMLST database (http://pubmlst.org/) (Jolley and Maiden, 2010). Phylogenetic tree of catenated, aligned antigen-encoding gene sequences (Fig. 2) was constructed using ClonalFrame and phylogenetic trees were visualized on CLC Genomics workbench v7 (www.clcbio.com) and MEGA v5.2 (Tamura et al., 2011).

### 2.5 Single nucleotide polymorphism (SNP) analysis

Raw sequence reads of 23 W ST-11 isolates for which we have short read sequence data were aligned to the Hajj reference genome *M7124* using BWA v0.6 (Li and Durbin, 2009). Aligned reads were indexed, sorted and filtered using VCFtools v0.1 with default options. All insertions and deletions (indels), diploid SNPs and SNPs with phred-like quality score, Q < 30 were excluded. Hajj specific *SNPs* were defined as those SNPs found in all Hajj cluster strains, but not non Hajj-cluster strains. Hajj cluster strains were defined as all strains phylogenetically and antigenically very closely related to the Hajj clone. We determined which discriminatory SNPs were acquired by recombination as opposed to spontaneous mutation by phylogenetic and BLASTN analyses of genomic sequences adjacent each Hajj specific SNP.

Bioinformatics analyses were done on BioLinux 7 server (Field et al., 2006) and Windows 7 computing environments.

### 2.6 Multilocus sequence typing (MLST) and outer membrane protein (OMP) gene sequencing

MLST and OMP sequence typing of the *porA* VR1, VR2, and *fetA* VR gene fragments were performed as described in Supplementary Methods.

### 2.7 16S rRNA gene sequencing

PCR amplification and sequence analysis of the 16S rRNA genes were performed using modifications of published methods (Lemos et al., 2010, Sacchi et al., 2002) as described in Supplementary Methods.

### 2.8 Funding

This study was supported by a career development award to Dr. Harrison, NIAID (K24 AI52788) and by a grant from the University of Pittsburgh Department of Epidemiology Small Grants Program. This project has also been funded in part with federal funds from the National Institute of Allergy and Infectious Diseases, National Institutes of Health, Department of Health and Human Services under contract number HHSN272200900009C.

### 2.9 Role of funding source

Funding sources played no role in study design; in the collection, analysis, and interpretation of data; in the writing of the report; and in the decision to submit the paper for publication.

## 3 Results

The Hajj clone (strain ID: *M7124*), and six other W ST-11 strains that were sequenced using PacBio provided very high quality reference sequences (Supplementary Table 1), with excellent resolution of capsular genes and other highly repetitive genomic regions.

**Fig. 3:**
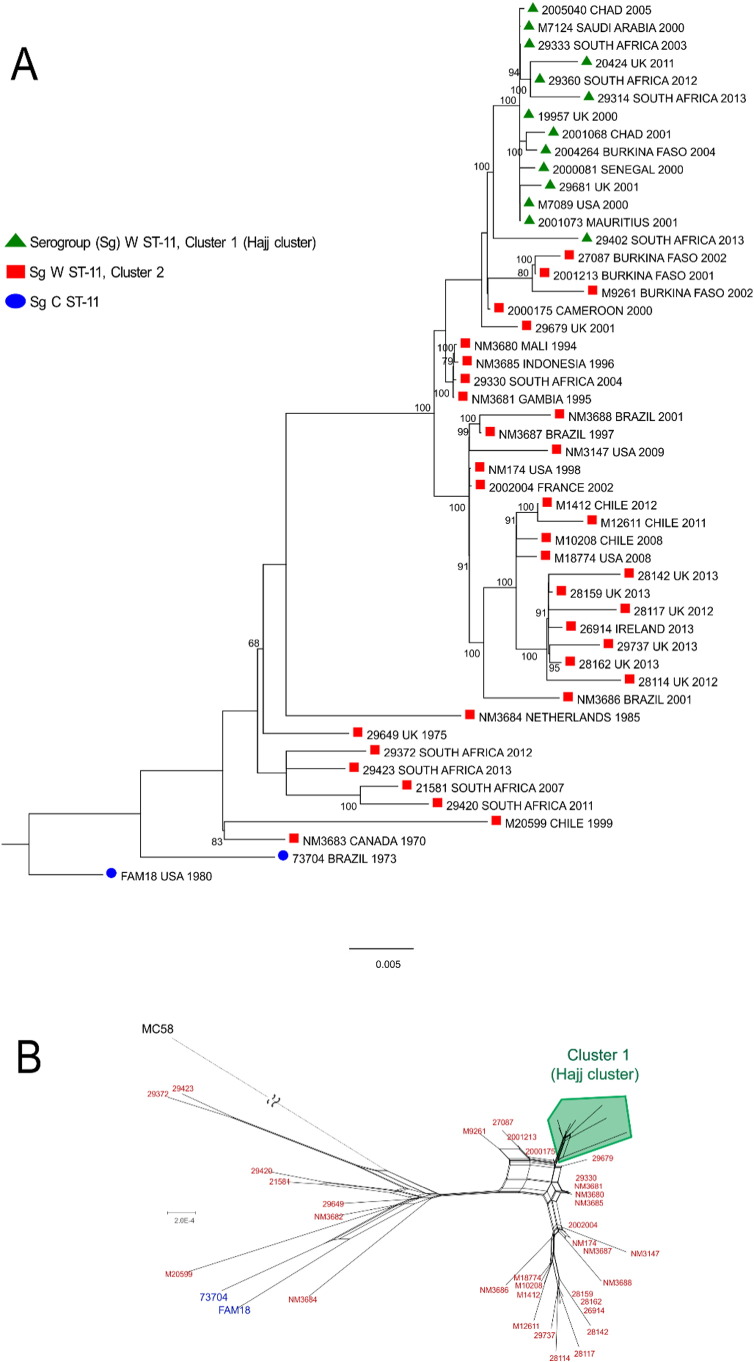
Core genome phylogenetic tree of serogroup W ST-11 and historical serogroup C ST-11 *N. meningitidis* strains (A). A maximum likelihood phylogenetic tree was constructed from aligned universally present genome sequences with 100 bootstrap iterations using the General Time Reversible model, gamma distribution of rate variation with invariant sites (GTR + Γ + I). Scale bar represents phylogenetic distance. Tree is rooted using the serogroup B reference strain *MC58* as outgroup (not shown). Bootstrap support values < 60% are not shown. SplitsTree phylogenetic network of generated from the alignment described above (B). In A-B above, several serogroup W ST-11 strains (Cluster 1) are phylogenetically very closely related to the Hajj clone reference strain *M7124* with 100% bootstrap support and a mean pairwise distance of to 0.000302; remaining W ST-11 strains (Cluster 2) are phylogenetically diverse with less than 60% bootstrap support and mean pairwise distance of 0.0017.

W ST-11 strains in this study were closely related to serogroup C ST-11 strains and clustered into two main groups based on genetic relatedness to the Hajj clone. Out of 270 total strains, 125 (46.3%) were most closely related to the Hajj clone based on antigen-encoding gene profiles (Fig. 2, Supplementary Table 2), presence of recombinant alleles (Table 1, Supplementary Fig. 1A–D) and whole genome phylogenetic analyses (Fig. 3A–B, Supplementary Fig. 2). We collectively refer to these strains as Cluster 1 (Hajj cluster). All strains in this cluster were isolated during or after the Hajj 2000 epidemic (post-Hajj strains). Remaining 145 strains were more heterogeneous having antigen-encoding gene, phylogenetic and SNP differences within key virulence genes compared to *M7124* and were collectively referred to as Cluster 2 strains. This cluster included both historical W ST-11 strains isolated before 2000 (pre-Hajj strains) and post-Hajj strains (2000–2013).

### 3.1 Phylogenetic analyses

Phylogenetic trees constructed using three different methods, Maximum Likelihood, SplitsTree and ClonalFrame consistently discriminated Cluster 1 from Cluster 2. Phylogenetic analyses of aligned whole genome sequences (Fig. 3A–B, Supplementary Fig. 2) and concatenated antigen-encoding gene sequences (Fig. 2) demonstrated that several strains (Cluster 1/Hajj cluster) were nearly identical to *M7124* suggesting that these strains were direct descendants of the Hajj clone. The other strains (Cluster 2) were phylogenetically diverse as shown by a bootstrap support of less than 60% for Cluster 2 compared to 100% among Cluster 1 strains (Fig. 3A) and a mean pairwise distance of 0.0017 among Cluster 2 compared to 0.0003 between Cluster 1 strains (Fig. 3B). Cluster 2 included a combination of recent and historical strains from the 1970s and 1980s that were most closely related to serogroup C ST-11 strains. SplitsTree phylogenetic network constructed from aligned core genome sequences support a clonal phylogenetic relationship between Cluster 1 strains (Fig. 3A–B, Supplementary Fig. 2). Also, a comparison of SNPs in 23 W ST-11 strains relative to *M7124* (Supplementary Table 2) showed that Cluster 1 strains had fewer SNP differences than Cluster 2 strains, which collectively support that Cluster 1 (Hajj cluster) strains represent clonal expansion within the W ST-11 lineage. Furthermore, these results demonstrate that many recent W ST-11 case clusters are more closely related to pre-Hajj sporadic strains and are unlikely to be direct descendants of the Hajj clone.

Genomic differences between the Cluster 1 and Cluster 2 strains were further explored by mapping the genomic locations of Hajj specific SNPs among 23 selected strains (Table 1). Of 48 Hajj specific SNPs, 46 were located in four genomic regions that ranged in size from 1·2–4·3 kb (Table 1). Phylogenetic and BLAST analyses suggest these genomic regions were acquired through homologous recombination (allelic exchange) [Table 1, Supplementary Fig. 1A–D]. Two of these recombinant regions encode known meningococcal antigens and/or virulence proteins – factor H binding protein (*fHbp*), nitric oxide reductase (*nor*), and nitrite reductase (*aniA*).

To explore the presence of these identified recombinant regions in all 270 strains in our collection, we queried nucleotide sequences from these recombinant regions in *M7124* against assembled contigs from all 270 genomes using BLASTn with 99% sequence identity and coverage. All four recombinant regions present in the Hajj clone were also found in 95·1% (118/124) of Cluster 1 strains. Six other Cluster 1 strains from South Africa 2005–2013 (*21583*, *29326*, *29336*, *29387*, *29402* and *29393*) had allelic profiles within one of four recombinant regions that were different from Hajj clone alleles (Supplementary Table 3). No Cluster 2 strain had a sequence closely matching any of the four recombinant regions associated with the Hajj clone (Table 2). These results demonstrate that these genomic regions represented areas with allelic replacement through recombination likely involving donor sequences from meningococci outside ST-11 lineage and commensal *Neisseria* species.

### 3.2 Antigen-encoding genes

#### 3.2.1 Cluster 1 (Hajj cluster)

The Hajj clone and 43% (116/270) of all W ST-11 strains in this study had an identical antigen-encoding gene profile: *porA* 1, *porB* 1, *fetA* 13, *nadA* 5, *nhba* 72 and *fHbp* 9 (Fig. 2, Supplementary Table 2). Strains with this profile were isolated from the meningitis belt in 2000–2005, Mauritius (2001), South Africa (2003–2013), United States (2000) and the UK (2000–2004, 2007, 2011). The Hajj clone antigen-encoding gene profile is characterized by a unique *fHbp* allele 9 (FHbp peptide 9, variant group 1/sub-family B). This *fHbp* allele was present only in Cluster 1 strains and not in sporadic pre-Hajj W ST-11 strains (Fig. 2, Supplementary Table 2). *fHbp* allele 9 was most likely acquired through allelic exchange within one of the four recombinant regions identified earlier (Supplementary Fig. 1 A-D). Nine other strains all isolated from South Africa in 2010–2013, differed only at the *fetA* gene compared to the Hajj clone, these strains were classified as Cluster 1 strains based on whole genome phylogenetic analyses and shared recombinant alleles with the Hajj clone.

#### 3.2.2 Cluster 2

Antigen-encoding gene profiles (Fig. 2, Supplementary Table 2) were more heterogeneous within Cluster 2 with two different alleles each for *nadA* and *nhba* and 8–11 different alleles for *porA*, *porB*, *fetA*, and *fHbp* genes. There were 33 different allelic combinations within Cluster 2 with 67.6% (98/145) having one of three predominant *porA*/*porB*/*fetA*/*nadA*/*nhba*/*fHbp* allelic profiles: 1/244/13/5/17/22, 1/311/13/5/17/160, and 1/1/13/3/17/22. None of the pre-Hajj strains shared an identical antigen-encoding gene profile with the Hajj clone. The *fHbp* locus was the most divergent between Cluster 1 and Cluster 2. The most common *fHbp* allele in Cluster 2 strains was allele 22 belonging to variant 2/family A present in 80.2% (117/145). None of the Cluster 2 strains had the Hajj clone *fHbp* gene allele. The remaining five antigen-encoding genes—*porA*, *porB*, *fetA*, *nadA* and *nhba* genes—were more closely related, with majority sharing 0–4 nucleotide differences, to the Hajj clone alleles (Fig. 2).

Strains in Cluster 2 were also geographically and temporally diverse, with W ST-11 strains isolated from the UK from 1970 to 1975 and Netherlands in 1985 being antigenically most closely related to the serogroup C ST-11 reference strain *FAM18* (Fig. 2, Supplementary Table 2). Five of nine strains from the meningitis belt isolated in 2000–2005 and 72.4% of 98 post-Hajj strains from South Africa (2003–2013) had the Hajj related antigen-encoding gene profile. In contrast, only one of three strains from the USA (2000, 2008–2009) and none of the five post-Hajj strains from Brazil and Chile had the Hajj related antigen-encoding gene profile (Supplementary Table 2). In the UK, strains with identical antigen-encoding gene profile to the Hajj clone predominated in 2000–2004 (97.8% of 45 strains) but were uncommon during 2005–2013 (3.3% of 90 strains, Supplementary Table 2). These data are consistent with antigenic diversification of W ST-11 strains presumably following an ancestral C to W ST-11 capsular switch, and subsequent emergence of the Hajj cluster from within Cluster 2 strains.

#### 3.2.3 16S Ribosomal RNA gene (16S) typing

16S rRNA type 31 was shared by the Hajj clone and six out of eight Cluster 1 strains while 14 of 16 Cluster 2 strains (87.5%) strains had 16S type 13. (Supplementary Table 2).

## 4 Discussion

In this study, we demonstrate that W ST-11 strains are closely related to serogroup C ST-11 *N. meningitidis* and likely arose from ancestral capsular switching events. We also demonstrate that the emergence of the Hajj clone in 2000 was caused by a meningococcal strain that was distinct from other circulating serogroup W ST-11 strains. This conclusion is supported by the congruent results from detailed whole genome phylogeny, antigen-encoding gene characterization, and identification of recombinant virulence gene alleles that were unique to the Hajj clone. This study clarifies the recent emergence of serogroup W ST-11 disease globally, which, based on previous limited genetic analyses, appeared to be caused by highly-related strains.

**Fig. 4:**
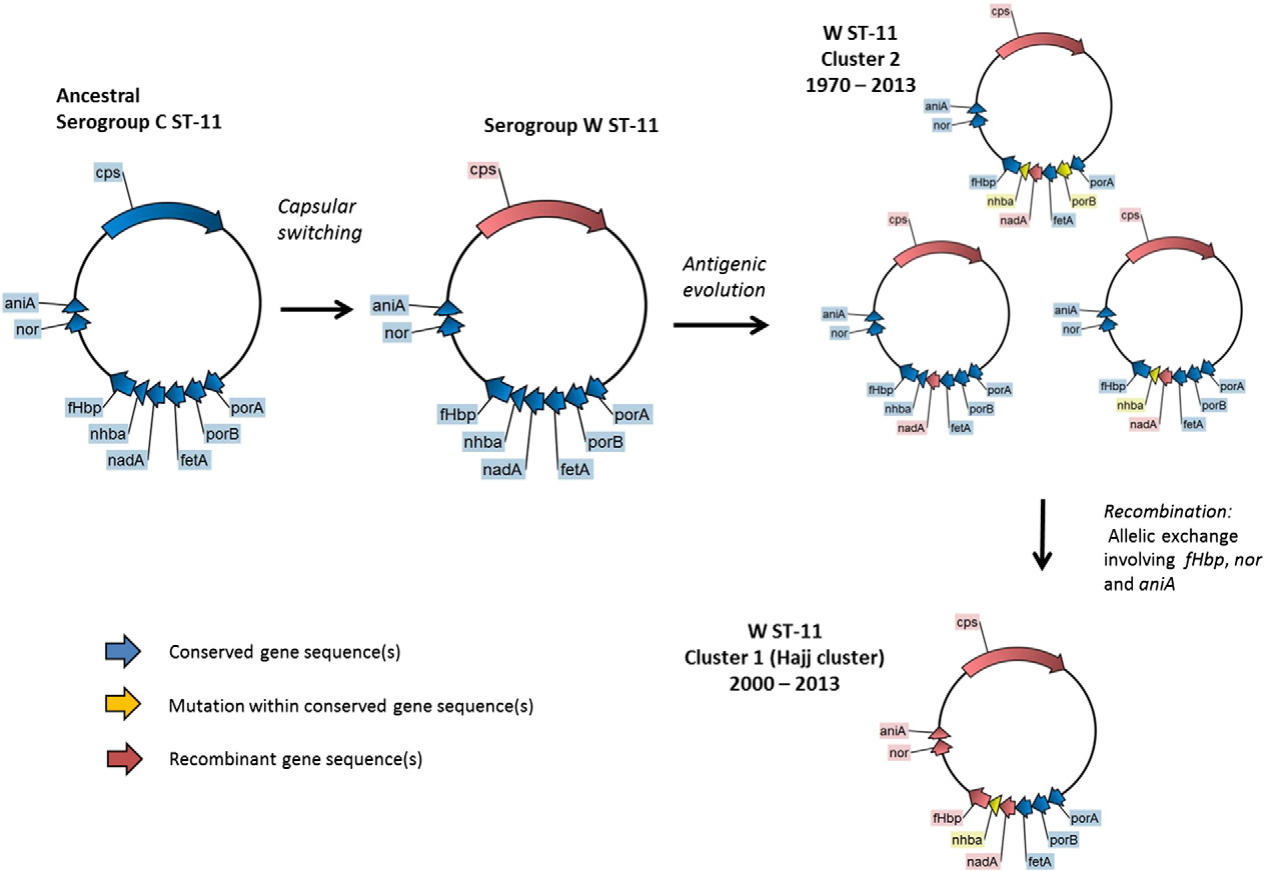
Genomic model of serogroup W ST-11 emergence and global spread. W ST-11 lineage likely emerged from an ancestral serogroup C ST-11 strain through ‘capsular switching’ – recombination within the capsular gene cluster (*cps*) involving donor sequences from a sporadic W strain and Y ST-23 (Mustapha et al., 2014) with subsequent antigenic diversification giving rise to sporadic W ST-11 strains (Cluster 2). The Hajj clone (Cluster 1) emerged through recombination within antigen-encoding and virulence genes *fHbp*, *nor* and *aniA*.

Based on these results and those from previous studies (Mayer et al., 2002, Mustapha et al., 2014), we propose a model whereby W ST-11 diverged from a C ST-11 ancestral strain through capsular switching before 1970 (Fig. 4). From the 1970s onward, W ST-11 strains disseminated to cause sporadic disease and case clusters globally (Cluster 2). Cluster 1 strains (Hajj cluster) evolved from sporadic Cluster 2 strains through allelic exchange within four recombinant regions two of which encode FHbp, nitric oxide reductase and nitrite reductase. This model supports global co-circulation of both Cluster 1 (Hajj cluster) and Cluster 2.

Sequence variation within the *fHbp* gene can potentially be used as a marker to identify the Hajj clone and closely related Cluster 1 strains. For example, a study of *fHbp* gene profiles from 47 endemic W ST-11 strains from 16 African countries isolated from 1980 to 2006 demonstrated that 34% of the strains shared the Hajj clone *fHbp* allele 9 (Pajon et al., 2011). Also, all W ST-11 strains with the Hajj clone *fHbp* allele 9 (variant family 1) were isolated after the Hajj 2000 epidemic while *fHbp* alleles belonging to variant family 2 and 3 were identified both before and after Hajj 2000. These findings are consistent with our results and support our evolutionary model.

Since 2001, Hajj related and endemic non Hajj W ST-11 strains have co-circulated across the meningitis belt. In 2002, the largest recorded epidemic of W ST-11 occurred in Burkina Faso with 12,000 cases and 1400 deaths (Koumare et al., 2007). It was generally believed that the Burkina Faso and other African W ST-11 epidemics were caused by direct spread of the Hajj clone. However, three Burkina Faso strains from 2001 to 2002 analyzed in this study all had antigen-encoding gene and other genomic markers consistent with non Hajj-cluster endemic W ST-11 strains. Additionally, Pajon et al (Pajon et al., 2011) reported that 76.5% (17/22) W ST-11 strains from Burkina Faso 2001–2003 had *fHbp* genotypes associated with endemic non Hajj strains. Epidemics of W ST-11 subsided in the meningitis belt from 2003 to 2009 despite persistence of small case clusters but resurfaced in 2010–2013 (Collard et al., 2010, Novak et al., 2012). Detailed antigenic and genomic characterization of more recent W ST-11 strains from the meningitis belt will be needed to monitor the continued evolution of the Hajj clone and endemic W ST-11 strains.

Outside the meningitis belt, serogroup W strains accounted for 62% of all invasive meningococcal disease strains in South Africa in 2005 compared to 5% in 2000, with 93% of W strains belonging to ST-11 lineage (Mothibeli et al., 2011). Our study reveals that 71·4% of 98 South African W ST-11 strains from 2003 to 2013 belonged to the Hajj cluster. These results are consistent with the finding that 85% of invasive W ST-11 strains isolated in South Africa in 2005 had the Hajj-related *fHbp* allele 9 (Mothibeli et al., 2011). Taken together, these data show that the Hajj cluster strains were predominant in South Africa. In the UK, Hajj related W ST-11 strains predominated in 2000–2004 but were replaced by endemic non Hajj strains thereafter (Valenzuela et al., 2013). Likewise, our results suggest that the small case clusters of W ST-11 in the United States 2008–2009 (Doyle et al., 2010), and larger clusters in south Brazil 2003–2005 (Lemos et al., 2010), and Chile 2010–2012 (Barra et al., 2013) represent the local spread of endemic strains with no evidence of direct spread of the Hajj clone.

16S ribosomal RNA gene sequencing was previously the most discriminatory test for differentiating the Hajj clone, which exhibited 16S type 31 compared to type 13 and 14 in sporadic W ST-11 strains (Mayer et al., 2002), However, our data demonstrate that some strains linked to the Hajj outbreak contained novel 16S alleles.

There are several hypotheses that could explain the emergence of the Hajj clone and subsequent W ST-11 outbreaks worldwide (Kelly and Pollard, 2003). FHbp is a major meningococcal antigen and a virulence determinant that is a component of vaccines developed for protection against serogroup B strains. The introduction of a novel FHbp antigenic type into an immunologically naïve population may have played a part in the emergence of the Hajj clone. In support of this hypothesis, the *fHbp* allele 9 unique to the Hajj related strains, belongs to variant group 1/sub-family B and has limited immunologic cross reactivity with variant groups 2 and 3/sub-family A, which were prevalent among Cluster 2 strains (Granoff, 2010, Beernink et al., 2009). Similarly, antigenic shift was associated with increases in serogroup C and serogroup Y meningococcal disease in the U.S. in the 1990s (Harrison et al., 2006).

Alternatively, the genomic changes we observed in the Hajj clone may be associated with increased virulence. For example, the nitrite reductase (*aniA*) gene — encoding a major outer membrane copper-containing protein, and the nitric oxide reductase (*nor*, sometimes referred to as *norB*) gene are both essential for overcoming oxidative stress and resistance to phagocytic lysis by macrophages (Anjum et al., 2002). Also, *N. meningitidis* lacking the *nor* gene have been shown to survive poorly in human nasopharyngeal tissue (Stevanin et al., 2005). Together, these data suggest that allelic variation in key virulence determinants may have a potential contribution to W ST-11 emergence. Then again, the genomic events (allelic exchange within *fHbp*, *nor* and *aniA*) observed could simply be markers of other, unidentified, genomic events that lead to changing epidemiologic behavior of W ST-11.

This study provides increasing evidence on the role of recombination in the emergence and persistence of meningococcal lineages and demonstrates the role of recombinant gene alleles in molecular epidemiologic typing of meningococcal isolates. We also add to the body of evidence showing the suitability of *fHbp* gene sequencing for routine meningococcal surveillance (Toros et al., 2014).

A limitation of this study is incomplete data as a result of variations in meningococcal disease surveillance by country and over the study period particularly in the 1970s-1990s. Historically, a large majority of ST-11 strains expressed serogroup C capsule with a significant minority of isolates expressing both B and W capsules. On the Neisseria PubMLST database (http://pubmlst.org/neisseria/), 78% of 665 ST-11 strains isolated in 1960–1999 expressed C capsule, while serogroups B and W each accounted for 10.4% of strains in the same period. Although our data suggest that the W ST-11 lineage diverged from an ancestral serogroup C ST-11 strain, the possibility that W ST-11 arose from another serogroup cannot be ruled out.

In summary, this study describes evidence of an ancestral capsular switching event and a model for the emergence, persistence and global spread of W ST-11 strains that are highly related to the Hajj 2000 outbreak strain. These data also demonstrate the co-circulation of W ST-11 strains that are phylogenetically and antigenically distinct from the Hajj clone and still cause disease in the African meningitis belt and globally. The emergence of the Hajj clone may have occurred because of the recent acquisition of a distinct antigen-encoding gene profile and genetic variations in meningococcal virulence genes.

## Author contributions

Conception of study hypothesis, aims and analytic plans: MMM, JWM, JGL, NLH, LHH. Acquisition and molecular characterization of meningococcal isolates: JWM, JOF, APSL, XW, LWM, LHH. Meningococcal genome sequencing, data analysis and interpretation: MMM, JWM, MGK, APSL, JOF, JCDH, XW, LWM, JGL, NLH, LHH. Initial draft and revision of study manuscript: MMM, JWM, MGK, JCDH, XW, LWM, JGL, NLH, LHH. Read and approved final manuscript: MMM, JWM, MGK, JOF, APSL, JCDH, XW, LWM, JGL, NLH, LHH.

## Declaration of interests

Dr. Harrison reports grants and personal fees from Sanofi Pasteur, personal fees from GSK, personal fees from Merck, personal fees from Novartis, personal fees from Pfizer, outside the submitted work; and all relationships with industry were terminated before I became a voting member of the Advisory Committee on Immunization Practices on July 1, 2012. Dr. Lemos reports travel grants and personal fees from Novartis, personal fees from Sanofi Pasteur, travel grants from GSK, outside the submitted work. Other co-authors have no interests to declare.

## Figures and Tables

**Table 1:** Homologous recombination regions associated with Hajj specific SNPs. Start and end indicate up- and downstream recombination breakpoints. Numbers and annotations are relative to the Hajj clone reference strain, *M7124*. Known virulence genes are shown in bold. No. of SNPs — Number of highly discriminatory SNPs within genomic region. Closest match — Closest match based on BLASTn query on PubMLST database (http://pubmlst.org/neisseria/).

	Genomic location of Hajj specific SNPs					
Recombinant region	Start	End	Size (kb)	No. of SNPs	Annotations	Closest match
**1**	329041	330204	1.2	5	Phosphopantetheine adenylyltransferase, *coaD*; ribosomal large subunit pseudouridine synthase D	*Neisseria cinerea*
**2**	628712	632970	4.3	13	Fructose-bisphosphate aldolase, *fba*; factor H binding protein, *fHbp*; glycoprotease family protein; ribosomal-protein-alanine acetyltransferase, *rimI*; uracil DNA glycosylase; Orotate phosphoribosyltransferase, *pyrE*; MJ0042 family finger-like domain protein; amino-acid N-acetyltransferase, *argA*	*Neisseria meningitidis* serogroup B ST-639 (cc32)
**3**	896826	900840	4.0	13	Argininosuccinate lyase, *argH*; UTP-Glucose-1-phosphate uridyl transferase, *galU*; purine NTP pyrophosphatase, *rdgB*; dATP pyrophosphohydrolase, *ntpA*; inorganic pyrophosphatase, *ppa*	*Neisseria spp.* ST-6263 (cc -)
**4**	1844312	1846343	2.0	15	Nitric oxide reductase, *nor*; nitrite reductase, *aniA*	*Neisseria meningitidis* ST-461 (cc461)

## References

[bb0005] Aguilera J.F., Perrocheau A., Meffre C., Hahne S., Group W.W. (2002). Outbreak of serogroup W135 meningococcal disease after the Hajj pilgrimage, Europe, 2000. Emerg. Infect. Dis..

[bb0010] Anjum M.F., Stevanin T.M., Read R.C., Moir J.W. (2002). Nitric oxide metabolism in *Neisseria meningitidis*. J. Bacteriol..

[bb0015] Aziz R.K., Bartels D., Best A.A. (2008). The RAST server: rapid annotations using subsystems technology. BMC Genomics.

[bb0020] Barra G.N., Araya P.A., Fernandez J.O. (2013). Molecular characterization of invasive *Neisseria meningitidis* strains isolated in Chile during 2010–2011. PLoS One.

[bb0025] Beernink P.T., Caugant D.A., Welsch J.A., Koeberling O., Granoff D.M. (2009). Meningococcal factor H-binding protein variants expressed by epidemic capsular group A, W-135, and X strains from Africa. J. Infect. Dis..

[bb0030] Bryant D., Moulton V. (2004). Neighbor-net: an agglomerative method for the construction of phylogenetic networks. Mol. Biol. Evol..

[bb0035] Capecchi B., Adu-Bobie J., Di Marcello F. (2005). Neisseria meningitidis NadA is a new invasin which promotes bacterial adhesion to and penetration into human epithelial cells. Mol. Microbiol..

[bb0040] Chevreux B., Pfisterer T., Drescher B. (2004). Using the miraEST assembler for reliable and automated mrna transcript assembly and snp detection in sequenced ESTs. Genome Res..

[bb0045] Chin C.S., Alexander D.H., Marks P. (2013). nonhybrid, finished microbial genome assemblies from long-read SMRT sequencing data. Nat. Methods.

[bb0050] Chiou C.S., Liao J.C., Liao T.L. (2006). Molecular epidemiology and emergence of worldwide epidemic clones of *Neisseria meningitidis* in Taiwan. BMC Infect. Dis..

[bb0055] Collard J.M., Maman Z., Yacouba H. (2010). Increase in *Neisseria meningitidis*serogroup W135, Niger, 2010. Emerg. Infect. Dis..

[bb0060] Darling A.E., Mau B., Perna N.T. (2010). ProgressiveMauve: multiple genome alignment with gene gain, loss and rearrangement. PLoS One.

[bb0065] Decosas J., Koama J.B. (2002). Chronicle of an outbreak foretold: meningococcal meningitis W135 in Burkina Faso. Lancet Infect. Dis..

[bb0070] Doyle T.J., Mejia-Echeverry A., Fiorella P. (2010). Cluster of serogroup W135 meningococci, southeastern Florida, 2008–2009. Emerg. Infect. Dis..

[bb0075] Efron A.M., Sorhouet C., Salcedo C., Abad R., Regueira M., Vazquez J.A. (2009). W135 invasive meningococcal strains spreading in South America: significant increase in incidence rate in Argentina. J. Clin. Microbiol..

[bb0080] Field D., Tiwari B., Booth T. (2006). Open software for biologists: from famine to feast. Nat. Biotechnol..

[bb0085] Fonkoua M.C., Taha M.K., Nicolas P. (2002). Recent increase in meningitis caused by *Neisseria meningitidis*serogroups A and W135, Yaounde, Cameroon. Emerg. Infect. Dis..

[bb0090] Granoff D.M. (2010). Review of meningococcal group B vaccines. Clin. Infect. Dis..

[bb0095] Guindon S., Dufayard J.F., Lefort V., Anisimova M., Hordijk W., Gascuel O. (2010). New algorithms and methods to estimate maximum-likelihood phylogenies: assessing the performance of PhyML 3.0. Syst. Biol..

[bb0100] Halperin S.A., Bettinger J.A., Greenwood B. (2012). The changing and dynamic epidemiology of meningococcal disease. Vaccine.

[bb0105] Harrison L.H., Jolley K.A., Shutt K.A. (2006). Antigenic shift and increased incidence of meningococcal disease. J. Infect. Dis..

[bb0110] Harrison L.H., Shutt K.A., Schmink S.E. (2010). Population structure and capsular switching of invasive *Neisseria meningitidis*Isolates in the pre-meningococcal conjugate vaccine era–United States, 2000–2005. J. Infect. Dis..

[bb0115] Holmes E.C., Urwin R., Maiden M.C. (1999). The influence of recombination on the population structure and evolution of the human pathogen *Neisseria meningitidis*. Mol. Biol. Evol..

[bb0120] Huson D.H., Bryant D. (2006). Application of phylogenetic networks in evolutionary studies. Mol. Biol. Evol..

[bb0125] Jolley K.A., Maiden M.C. (2010). BIGSdb: scalable analysis of bacterial genome variation at the population level. BMC Bioinf..

[bb0130] Katz L.S., Humphrey J.C., Conley A.B. (2011). *Neisseria* base: a comparative genomics database for *Neisseria meningitidis*. Database.

[bb0135] Kelly D., Pollard A.J. (2003). W135 in Africa: Origins, Problems and Perspectives. Travel Med. Infect. Dis..

[bb0140] Kong Y., Ma J.H., Warren K. (2013). Homologous recombination drives both sequence diversity and gene content variation in *Neisseria meningitidis*. Genome Biol. Evol..

[bb0145] Koumare B., Ouedraogo-Traore R., Sanou I. (2007). The first large epidemic of meningococcal disease caused by serogroup W135, Burkina Faso, 2002. Vaccine.

[bb0150] Ladhani S.N., Beebeejaun K., Lucidarme J. (2015). Increase in endemic Neisseria meningitidis capsular group W sequence type 11 complex associated with severe invasive disease in England and Wales. Clin. Infect. Dis..

[bb0155] Lapeyssonnie L. (1968). Comparative epidemiologic study of meningococcic cerebrospinal meningitis in temperate regions and in the meningitis belt in Africa. Attempt at synthesis. Med. Trop..

[bb0160] Lemos A.P., Harrison L.H., Lenser M., Sacchi C.T. (2010). Phenotypic and molecular characterization of invasive serogroup W135 *Neisseria meningitidis*strains from 1990 to 2005 in Brazil. J. Infect..

[bb0165] Li H., Durbin R. (2009). Fast and accurate short read alignment with Burrows-Wheeler transform. Bioinformatics.

[bb0170] MacNeil J.R., Medah I., Koussoube D. (2014). *Neisseria meningitidis*serogroup W, Burkina Faso, 2012. Emerg. Infect. Dis..

[bb0175] Maiden M.C., Bygraves J.A., Feil E. (1998). Multilocus sequence typing: aportable approach to the identification of clones within populations of pathogenic microorganisms. Proc. Natl. Acad. Sci. U. S. A..

[bb0180] Mayer L.W., Reeves M.W., Al-Hamdan N. (2002). Outbreak of W135 meningococcal disease in 2000: not emergence of a new W135 strain but clonal expansion within the electophoretic type-37 complex. J. Infect. Dis..

[bb0185] Mothibeli K.M., du Plessis M., von Gottberg A. (2011). Distribution of factor H binding protein beyond serogroup B: variation among five serogroups of invasive *Neisseria meningitidis* in South Africa. Vaccine.

[bb0190] Multi-Disease Surveillance Centre Ouagadougou RMS (2002–2015). Weekly feedback bulletin on cerebrospinal meningitis. http://www.who.int/csr/disease/meningococcal/epidemiological/en/index.html;.

[bb0195] Mustapha M., Mustapha J.W.M., Jorge O., Fernandez A.P.S., de Lemos X.W., Mayer L.W., Harrison L.H. (2014). Capsular Switching and Global Spread of *Neisseria meningitidis* Serogroup W, ST-11. International Pathogenic Neisseria Conferences, IPNC, 2014.

[bb0200] Novak R.T., Kambou J.L., Diomande F.V. (2012). Serogroup A meningococcal conjugate vaccination in Burkina Faso: analysis of national surveillance data. Lancet Infect. Dis..

[bb0205] Pajon R., Fergus A.M., Koeberling O., Caugant D.A., Granoff D.M. (2011). Meningococcal factor H binding proteins in epidemic strains from Africa: implications for vaccine development. PLoS Negl. Trop. Dis..

[bb0210] Russell J.E., Jolley K.A., Feavers I.M., Maiden M.C., Suker J. (2004). PorA variable regions of Neisseria meningitidis. Emerg. Infect. Dis..

[bb0215] Sacchi C.T., Whitney A.M., Reeves M.W., Mayer L.W., Popovic T. (2002). Sequence diversity of *Neisseria meningitidis* 16S rRNA genes and use of 16S rRNA gene sequencing as a molecular subtyping tool. J. Clin. Microbiol..

[bb0220] Seib K.L., Scarselli M., Comanducci M., Toneatto D., Masignani V. (2015). Neisseria meningitidis factor H-binding protein fHbp: a key virulence factor and vaccine antigen. Expert Rev. Vaccines.

[bb0225] Serruto D., Spadafina T., Ciucchi L. (2010). Neisseria meningitidis GNA2132, a heparin-binding protein that induces protective immunity in humans. Proc. Natl. Acad. Sci. U. S. A..

[bb0230] Stephens D.S. (2009). Biology and pathogenesis of the evolutionarily successful, obligate human bacterium *Neisseria meningitidis*. Vaccine.

[bb0235] Stevanin T.M., Moir J.W., Read R.C. (2005). Nitric oxide detoxification systems enhance survival of *Neisseria meningitidis* in human macrophages and in nasopharyngeal mucosa. Infect. Immun..

[bb0240] Swartley J.S., Marfin A.A., Edupuganti S. (1997). Capsule switching of *Neisseria meningitidis*. Proc. Natl. Acad. Sci. U. S. A..

[bb0245] Taha M.K., Achtman M., Alonso J.M. (2000). Serogroup W135 meningococcal disease in Hajj pilgrims. Lancet.

[bb0250] Taha M.K., Giorgini D., Ducos-Galand M., Alonso J.M. (2004). Continuing diversification of *Neisseria meningitidis* W135 as a primary cause of meningococcal disease after emergence of the serogroup in 2000. J. Clin. Microbiol..

[bb0255] Tamura K., Peterson D., Peterson N., Stecher G., Nei M., Kumar S. (2011). MEGA5: molecular evolutionary genetics analysis using maximum likelihood, evolutionary distance, and maximum parsimony methods. Mol. Biol. Evol..

[bb0260] Tanabe M., Nimigean C.M., Iverson T.M. (2010). Structural basis for solute transport, nucleotide regulation, and immunological recognition of Neisseria meningitidis PorB. Proc. Natl. Acad. Sci. U. S. A..

[bb0265] Thompson E.A., Feavers I.M., Maiden M.C. (2003). Antigenic diversity of meningococcal enterobactin receptor FetA, a vaccine component. Microbiology.

[bb0270] Toros B., Thulin Hedberg S., Jacobsson S., Fredlund H., Olcen P., Molling P. (2014). Surveillance of invasive Neisseria meningitidis with a serogroup Y update, Sweden 2010 to 2012. Euro Surveill..

[bb0275] Valenzuela M.T., Moreno G., Vaquero A. (2013). Emergence of W135 meningococcal serogroup in Chile during 2012. Rev. Med. Chil..

[bb0280] von Gottberg A., du Plessis M., Cohen C. (2008). Emergence of endemic serogroup W135 meningococcal disease associated with a high mortality rate in South Africa. Clin. Infect. Dis..

[bb0285] Vos M., Didelot X. (2009). A comparison of homologous recombination rates in bacteria and archaea. ISME J..

[bb0290] Zhou H., Liu W., Xu L. (2013). Spread of *Neisseria meningitidis*serogroup W clone, *China*. Emerg. Infect. Dis..

