# Supplementary material for: Genomic Epidemiology of Hypervirulent Serogroup W, ST-11 Neisseria meningitidis
Source: eBioMedicine. 2015 Sep 8;2(10):1447–55. doi: 10.1016/j.ebiom.2015.09.007 (PMC4634745; doi:10.1016/j.ebiom.2015.09.007)
Supplement: Supplementary file 1 — Supplementary material. [file mmc1.docx]

SUPPLEMENTARY METHODS

*DNA extraction:* Isolates for DNA extraction were subcultured from a single colony and grown overnight at 37°C with 5% CO_2_ on chocolate agar plates. DNA for Sanger sequencing was extracted by a NucliSENs easyMAG system (bioMérieux, Durham, NC) and by boiling a heavy suspension of organisms in 0.5mL of PBS. DNA for Illumina and Ion Torrent sequencing was extracted by using the DNeasy blood and tissue kit (QIAGEN, Valencia, CA). DNA for PacBio sequencing was extracted using the Easy-DNA gDNA Purification Kit (Invitrogen, Grand Island, NY).

*Multilocus sequene typing (MLST) and outer membrane protein (OMP) gene sequencing:*  For all 26 sequenced genomes PCR based MLST and OMP gene sequence typing of PorA VR1 and VR2, and FetA VR were performed as described.[^1^](#_ENREF_1) Sequence alleles and sequence types were determined by querying the PubMLST database. MLST and OMP results were compared to those obtained by querying assembled contigs on PubMLST database. MLST and OMP typing for public genomes were obtained by querying genome sequences against the PubMLST database.

*16S rRNA gene sequencing:* PCR amplification and sequence analysis of the 16S rRNA genes were performed using modifications of published methods.[^2^](#_ENREF_2)^,^[^3^](#_ENREF_3) Primers 8F and 1492R were used for amplification. All reactions were carried out in 50-μl volumes containing 1 μl of purified genomic DNA (~20 ng), 1× AmpliTaq Gold PCR buffer (15 mM Tris-HCl, pH 8.0, 50 mM KCl), 2.5 mM MgCl_2_, 0.2 mM each deoxynucleoside triphosphate, 0.2 μM of each primer, and 1.5 units AmpliTaq Gold DNA polymerase (Applied Biosystems, Foster City, CA). Cycling conditions were an initial denaturation step at 95°C for 5 min followed by 35 cycles of 95°C for 1 min, 50°C for 1 min and 72°C for 1.5 min, followed by a final extension step for 7 min at 72°C. PCR products were purified with Exo-Sap It (Affymetrix, Santa Clara, CA) and sequenced with BigDye Terminator v3.1 Cycle Sequencing Kit according to manufacturer’s instructions using previously described primers 8F, 1492R, 357, 530, 790, 981, 1968F and 1083F.[^2^](#_ENREF_2)^,^[^3^](#_ENREF_3) Sequence contigs were analyzed and compared to 16S type 13 and 31 by using DNAstar Lasergene SeqMan Pro software (v.11.2; DNAstar, Madison, WI).

*SNP-based whole genome phylogenetic tree* (Supplemetary Figure 2A) was generated by aligning sequence reads of 153 N. meningitidis genome sequences (Supplementary Table 4) against *M7124* genome. Aligned reads were sorted and filtered as descrided in Methods. 140,903 SNP positions were concatenated and a maximum likelihood tree generated under General Time Reversible model, gamma distribution of rate variation with invariant sites (GTR+Γ+I).

For all maximum likelihood phylogenetic analyses, model of evolution with the lowest BIC score was selected using Modeltest program on Mega v5.2.

*Quality assessment:* Several steps were taken during isolate selection, genome assembly, and data analyses to assure data quality and consistency of results. For all 26 newly sequenced genomes at least three iterations of genome assemblies were run and the the assembly with the best assembly quality metrics (low assembly gaps, high average median contig size and larger total genome size) is selected. Assembly quality was further assessed by comparing newly sequenced genomes to both group C ST-11 reference strain, *FAM18* and *M7124* using multiple genome alignments on Mauve v2.3. In addition, read mapping and SNP analyses were used to compare data obtained from different platforms for re-sequenced isolates. No major discrepancies in the form of insertion-deletion, translocation or inconsistent genome sizes were identified.

Three isolates that originally had IonTorrent data were re-sequenced using PacBio to assess whether the relatively large number of assembly gaps obtained from IonTorrent affected phylogenetic clustering of these isolates. Re-sequencing did not change the phylogenetic relationships between strains in our study (data not shown). To ascertain that only good quality genome assemblies were included from PubMLST database, genomes that had incomplete data at one of six antigen gene alleles were excluded from the study.

For whole genome phylogenetic analyses, isolates were selected to capture geographic and antigen gene allelic diversity of the entire set of 270 isolates. Among isolates sharing similar allelic and geographic profiles, one with the best genome assembly parameters was selected as a representative strain. ‘Iterative refinement’ option was selected to improve genome alignment accuracy while poorly aligned genomic regions were excluded by including only core genome segments that were conserved in all aligned genomes and were at least 500bp in size. The resultant core genome Maximum Likelihood phylogenetic tree remained unchanged after increasing the minimum alignment block size to 2000bp and/or removing very small alignment gaps not addressed by Mauve using GBlocks (results not shown). SNP analysis was limited to 20 genomes that had short read sequencing data (Illumina, IonTorrent, 454). PacBio reads were not used for SNP calling because the tools we used (bwa, samtools) were not designed to handle long read sequence data.

Finally, we compared results of separate phylogenetic trees constructed using ClonalFrame, SptitsTree and Maximum Likelihood to allelic profiles of antigen encoding genes. Concordance between these data and available epidemiologic information for these isolates provided further reassurance of the validity of these results.

REFERENCES

1. Feavers IM, Gray SJ, Urwin R, et al. Multilocus sequence typing and antigen gene sequencing in the investigation of a meningococcal disease outbreak. *Journal of clinical microbiology* 1999; **37**(12): 3883-7.

2. Lemos AP, Harrison LH, Lenser M, Sacchi CT. Phenotypic and molecular characterization of invasive serogroup W135 *Neisseria meningitidis* strains from 1990 to 2005 in Brazil. *The Journal of Infection* 2010; **60**(3): 209-17.

3. Sacchi CT, Whitney AM, Reeves MW, Mayer LW, Popovic T. Sequence diversity of *Neisseria meningitidis* 16S rRNA genes and use of 16S rRNA gene sequencing as a molecular subtyping tool. *Journal of clinical microbiology* 2002; **40**(12): 4520-7.

4. Chin CS, Alexander DH, Marks P, et al. Nonhybrid, finished microbial genome assemblies from long-read SMRT sequencing data. *Nature Methods* 2013; **10**(6): 563-9.

5. Chevreux B, Pfisterer T, Drescher B, et al. Using the miraEST assembler for reliable and automated mRNA transcript assembly and SNP detection in sequenced ESTs. *Genome Research* 2004; **14**(6): 1147-59.

6. Aziz RK, Bartels D, Best AA, et al. The RAST Server: rapid annotations using subsystems technology. *BMC Genomics* 2008; **9**: 75.

7. Didelot X, Falush D. Inference of bacterial microevolution using multilocus sequence data. *Genetics* 2007; **175**(3): 1251-66.

8. Vos M, Didelot X. A comparison of homologous recombination rates in bacteria and archaea. *The ISME Journal* 2009; **3**(2): 199-208.

9. Field D, Tiwari B, Booth T, et al. Open software for biologists: from famine to feast. *Nature Biotechnology* 2006; **24**(7): 801-3.

Supplementary Figure 1 A


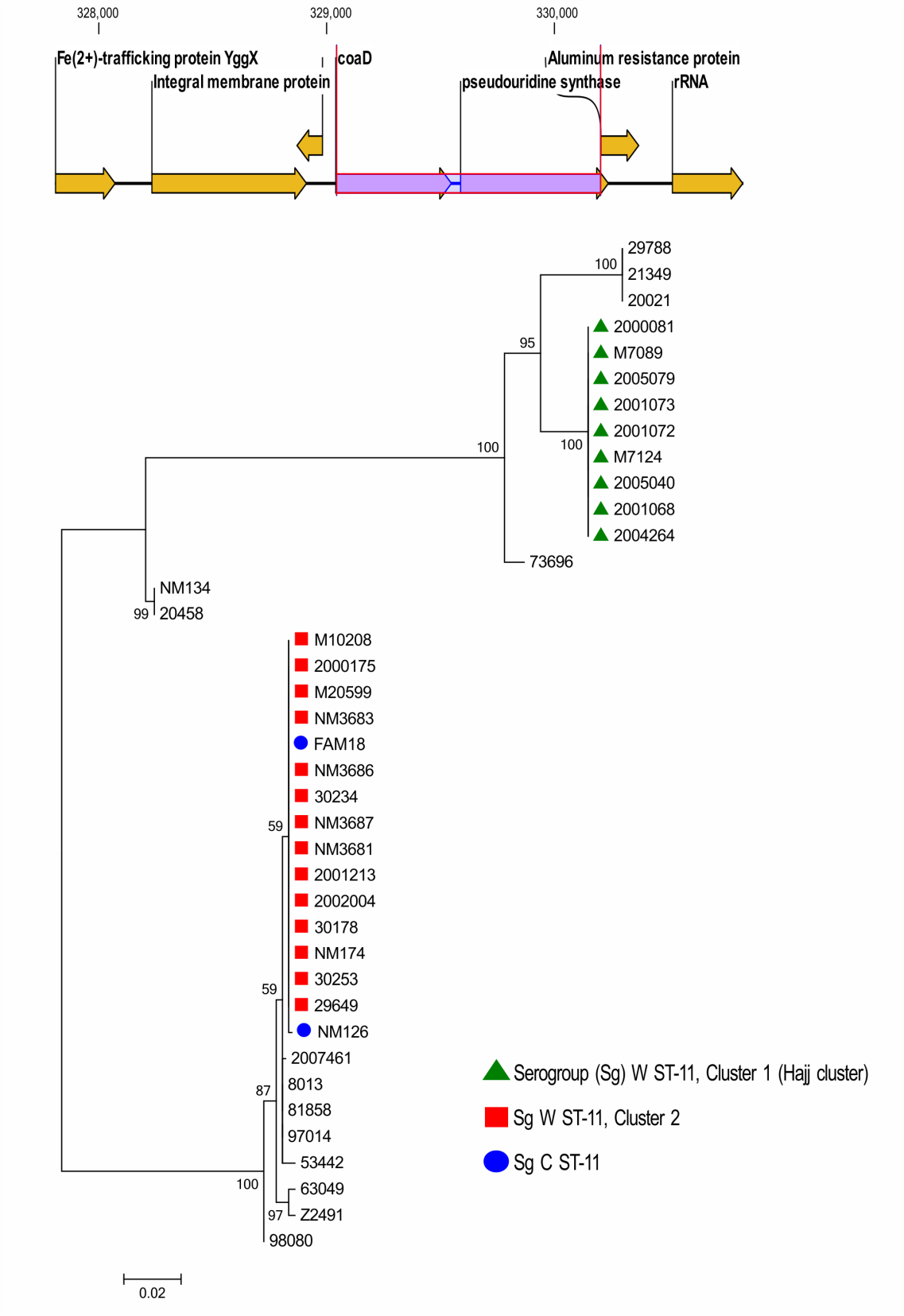


Supplementary Figure 1 B


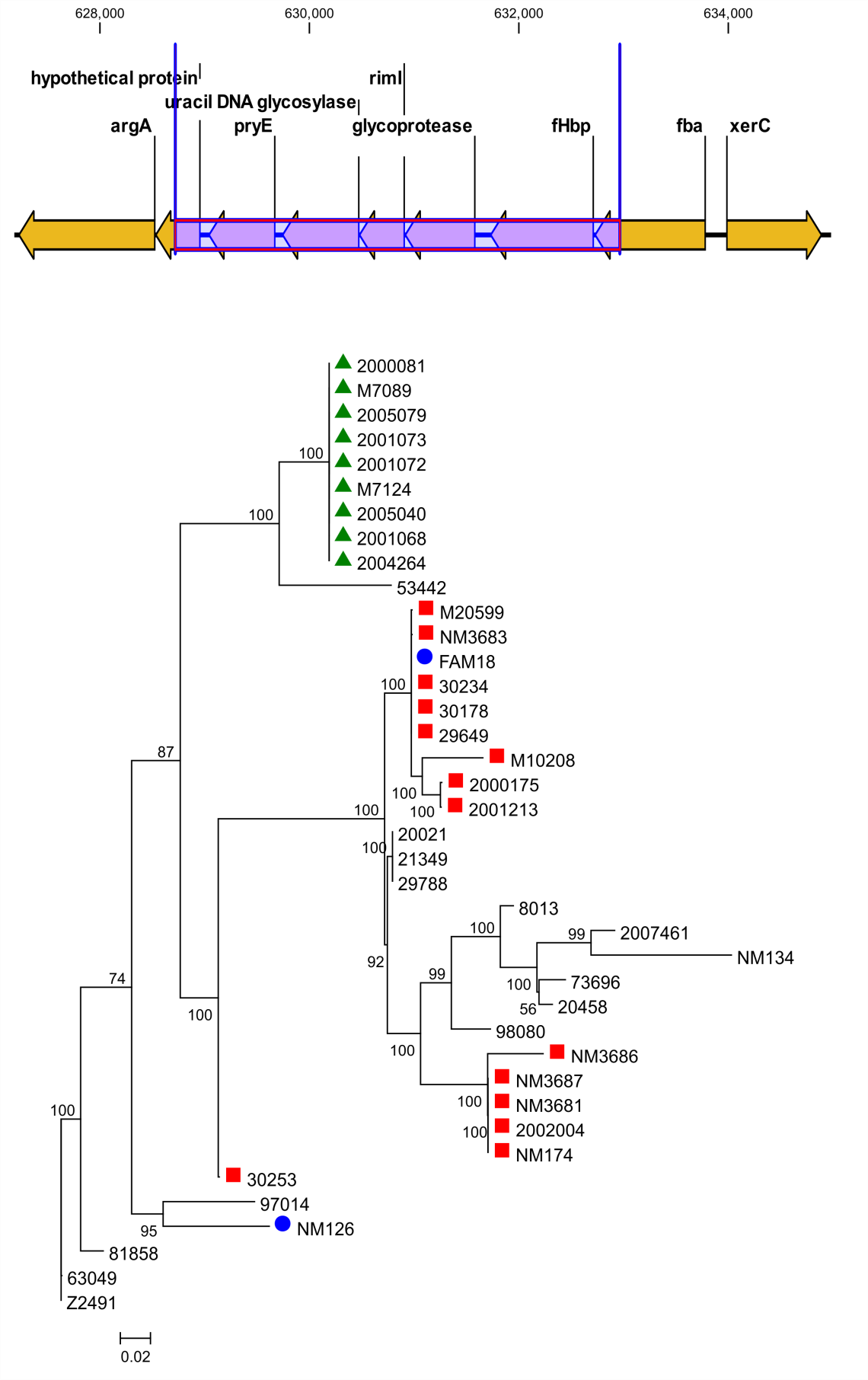


Supplementary Figure 1C


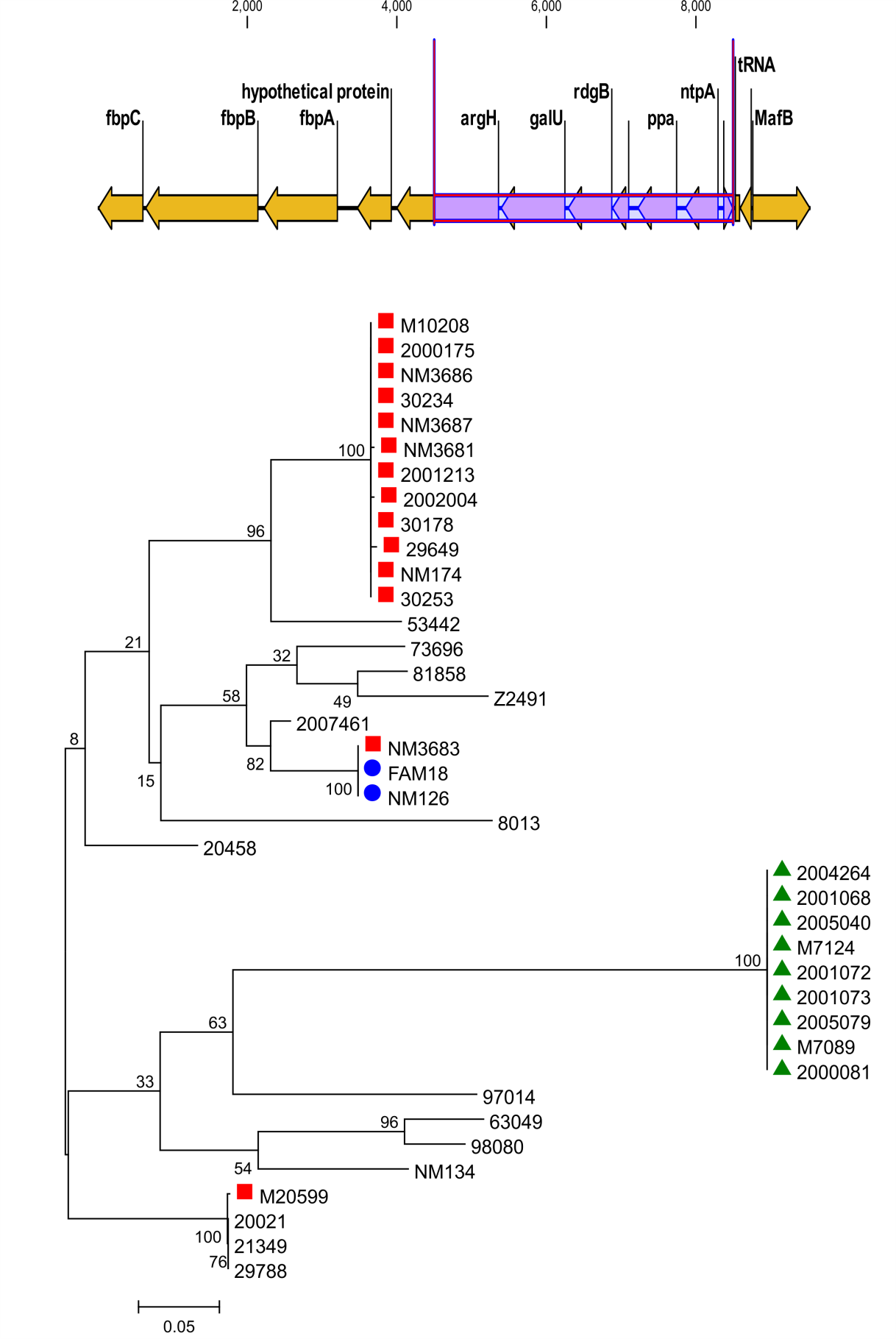


Supplementary Figure 1D


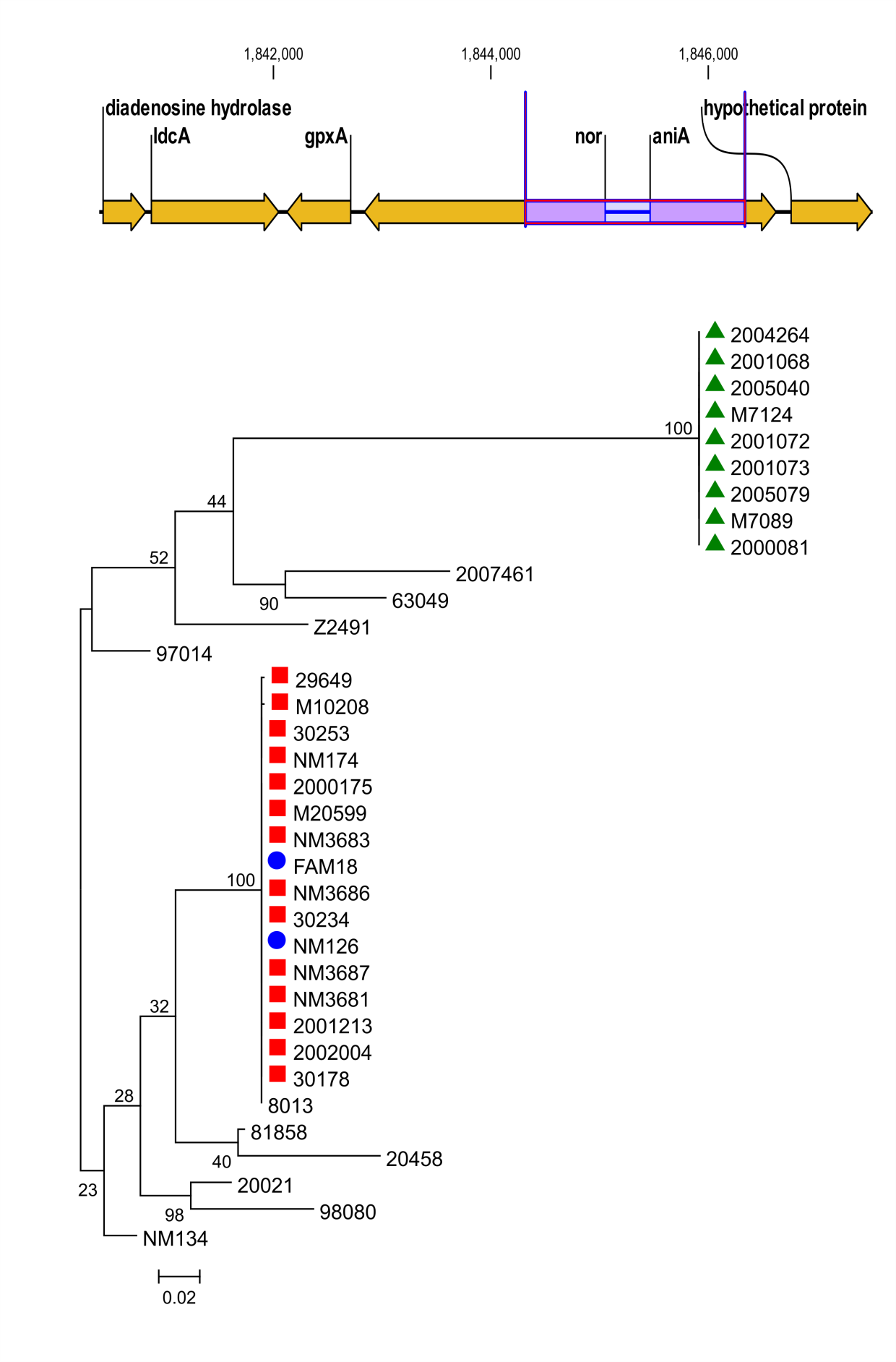


Supplementary Figure 2


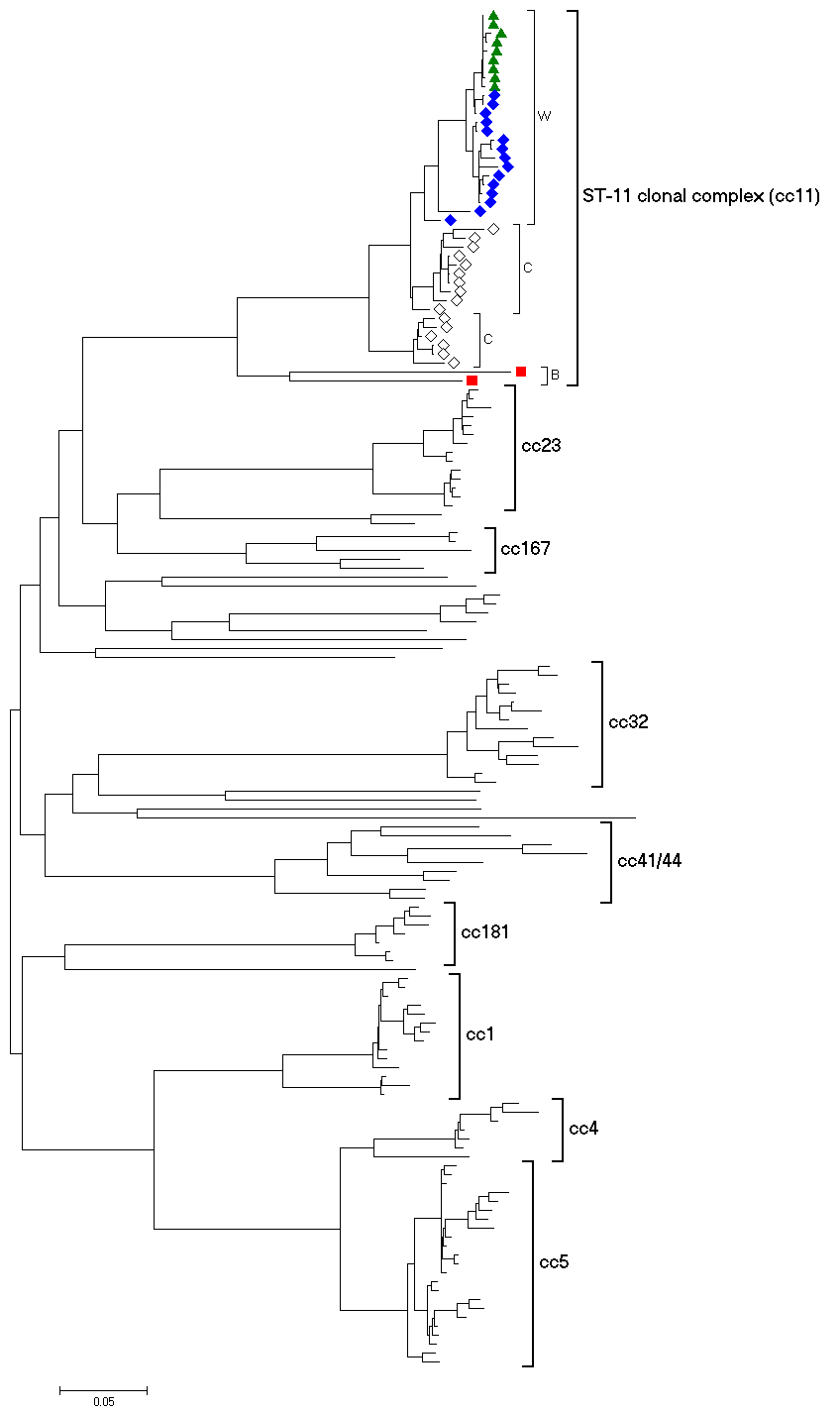


**Supplementary Table 1: Genome assembly statistics for 26 meningococcal genomes sequenced in this study.**

| **Strain ID** | **Sequencing technology** | **GC content** | **Coverage** | **Assembly method** | **Number of contigs** | **GenBank**  **Accession No.** | **Genome size** | **Largest contig size** | **Median contig size** | **Smallest contig size** | **N50**^1^ | **N90**^2^ |
| --- | --- | --- | --- | --- | --- | --- | --- | --- | --- | --- | --- | --- |
| M7124 | PacBio^3^ | 51.7 | 130.9 | HGAP v2.0 | 1 | CP009419 | 2179483 | - | - | - | - | - |
| NM3686 | PacBio | 51.7 | 173.0 | HGAP v2.0 | 1 | CP009418 | 2195266 | - | - | - | - | - |
| NM3687 | PacBio | 51.6 | 219.8 | HGAP v2.0 | 10 | JRET00000000 | 2223230 | 1610344 | 47059 | 4073 | 1609697 | 103326 |
| NM3681 | PacBio | 51.7 | 62.1 | HGAP v2.0 | 2 | JREU00000000 | 2217098 | 1380815 | 415099 | 6085 | 1380815 | 12054 |
| NM3682 | PacBio | 51.7 | 60.0 | HGAP v2.0 | 1 | CP009420 | 2185359 | - | - | - | - | - |
| M10208 | PacBio | 51.7 | 55.0 | HGAP v2.0 | 1 | CP009422 | 2183230 | - | - | - | - | - |
| M20599 | PacBio | 51.8 | 60.0 | HGAP v2.0 | 13 | JRFG00000000 | 2258407 | 1053953 | 19069 | 4563 | 516162 | 150267 |
| M1412 | Ion Torrent^4^ | 51.7 | 62.6 | Mira v3.9 | 320 | JRFH00000000 | 2110370 | 39255 | 4109 | 504 | 12232 | 3559 |
| M12611 | Ion Torrent | 51.8 | 70.8 | Mira v3.9 | 698 | JRFI00000000 | 1914608 | 13374 | 2130 | 10001 | 3305 | 1405 |
| M7089 | Illumina+454^5^ | 51.7 | 32.0 | Celera v7.0 | 31 | ANRN00000000 | 2174132 | 280052 | 51414 | 1714 | 133850 | 49971 |
| 2001213 | Illumina^6^ | 51.9 | 54.7 | Celera v7.0 | 125 | APUD00000000 | 2145367 | 107785 | 10070 | 516 | 29037 | 8528 |
| 2004264 | Illumina | 52.0 | 57.6 | Celera v7.0 | 132 | APUE00000000 | 2223810 | 87880 | 11580 | 179 | 27158 | 8339 |
| 2005079 | Illumina | 52.0 | 61.6 | Celera v7.0 | 132 | APUG00000000 | 2121623 | 78339 | 10799 | 136 | 27213 | 8691 |
| 2005040 | Illumina | 52.0 | 62.4 | Celera v7.0 | 133 | APUI00000000 | 2156155 | 169873 | 10487 | 146 | 27155 | 9111 |
| 2001072 | Illumina | 51.9 | 75.5 | Celera v7.0 | 131 | APTY00000000 | 2157792 | 124299 | 10473 | 120 | 26510 | 8615 |
| 2001073 | Illumina | 51.9 | 79.4 | Celera v7.0 | 137 | APTZ00000000 | 2143674 | 86269 | 10192 | 118 | 28492 | 8200 |
| 2002004 | Illumina | 51.9 | 55.9 | Celera v7.0 | 137 | APUJ00000000 | 2256966 | 68617 | 12347 | 133 | 26724 | 8625 |
| 2000081 | Illumina | 51.9 | 60.4 | Celera v7.0 | 140 | APUA00000000 | 2180008 | 63594 | 10344 | 101 | 26618 | 8225 |
| NM174 | Illumina+454 | 51.7 | 43.1 | Celera v7.0 | 19 | ANRP00000000 | 2180278 | 771479 | 80215 | 1914 | 161674 | 67157 |
| 2000175 | Illumina | 51.9 | 51.4 | Celera v7.0 | 134 | APUF00000000 | 2233577 | 93253 | 11468 | 224 | 25838 | 8910 |
| 2001068 | Illumina | 51.9 | 70.2 | Celera v7.0 | 136 | APUH00000000 | 2214688 | 94883 | 11879 | 648 | 24221 | 8778 |
| NM3680 | Ion Torrent | 51.8 | 63.2 | Mira v3.9 | 250 | JSAR00000000 | 2101022 | 65014 | 5616 | 507 | 15072 | 4516 |
| NM3684 | Ion Torrent | 51.7 | 64.1 | Mira v3.9 | 261 | JSAS00000000 | 2132590 | 55229 | 5131 | 497 | 16290 | 4425 |
| NM3685 | Ion Torrent | 51.7 | 47.3 | Mira v3.9 | 280 | JSAT00000000 | 2122125 | 49178 | 4782 | 497 | 13937 | 3925 |
| NM3688 | Ion Torrent | 51.6 | 63.7 | Mira v3.9 | 249 | JSAU00000000 | 2165310 | 59342 | 5196 | 501 | 17223 | 4867 |
| NM3147 | Illumina | 52.0 | 81.06 | Celera v7.0 | 136 | APTX00000000 | 2256933 | 94650 | 10744 | 101 | 29024 | 8163 |

^1^N50: 50% of the entire genome is contained in contigs equal to or larger than this value.

^2^N90: 90% of the entire genome is contained in contigs equal to or larger than this value; HGAP: Hierarchical genome assembly process.

^3^Pacific BioSciences RS II sequencing (www.pacificbiosciences.com/)

^4^Life Technologies Ion Torrent PGM sequencing (www.lifetechnologies.com)

^5^Roche 454 sequencing (www.454.com)

^6^Paired end Illumina MiSEQ (http://www.illumina.com/)

**Supplementary Table 2: Epidemiologic and genetic characteristics of 270 serogroup W ST-11 strains.***

| **Strain ID** | **Country** | **Year** | **Sequenced by** | **ST**^1^ | **CC**^2^ | **PorA VR1^3^** | **PorA VR2** | **FetA VR**^4^ | **Phylogenetic cluster**^5^ | ***porA*** | ***porB*** | ***fetA*** | ***nadA*** | ***nhba*** | ***fHbp*** | **16S**^6^ | **SNPs**^7^ |
| --- | --- | --- | --- | --- | --- | --- | --- | --- | --- | --- | --- | --- | --- | --- | --- | --- | --- |
| **M7124** | Saudi | 2000 | This study | 11 | 11 | 5 | 2 | F1-1 | Cluster 1 (Hajj cluster) | 1 | 1 | 13 | 5 | 72 | 9 | 31 | Reference |
| **NM3688** | Brazil | 2001 | This study | 11 | 11 | 5 | 2 | F1-1 | Cluster 2 | 1 | UA^10^ | 13 | 5 | 17 | 160 | 13 | 1827 |
| **NM3687** | Brazil | 2001 | This study | 11 | 11 | 5 | 2 | F1-1 | Cluster 2 | 1 | UA | 13 | 5 | 17 | UA | 13 | 1551 |
| **2001213** | Burkina Faso | 2001 | This study | 11 | 11 | 5 | 2 | F1-1 | Cluster 2 | 1 | 1 | 13 | 5 | 17 | 62 | 13 | 775 |
| **2000175** | Cameroon | 2000 | This study | 11 | 11 | 5 | 2 | F1-1 | Cluster 2 | 1 | 1 | 13 | 5 | 72 | UA | 13 | 447 |
| **M20599** | Chile | 1999 | This study | 11 | 11 | 5 | 10 | F3-6 | Cluster 2 | UA | 425 | UA | 3 | 17 | 22 | 13 | N.D.^11^ |
| **M10208** | Chile | 2008 | This study | 11 | 11 | 5 | 2 | F1-1 | Cluster 2 | 1 | 244 | 13 | 5 | 17 | 752 | 13 | N.D. |
| **NM3681** | Gambia | 1995 | This study | 11 | 11 | 5 | 2 | F1-1 | Cluster 2 | 1 | 311 | 13 | 5 | 17 | 160 | 13 | N.D. |
| **NM3685** | Indonesia | 1996 | This study | 11 | 11 | 5 | 2 | F1-1 | Cluster 2 | 1 | 311 | 13 | 5 | 17 | 160 | 13 | 1360 |
| **NM3680** | Mali | 1994 | This study | 11 | 11 | 5 | 2 | F1-1 | Cluster 2 | 1 | 311 | 13 | 5 | 17 | 160 | 13 | 1206 |
| **NM3682** | Scotland | 1970 | This study | 11 | 11 | 5 | 2 | F1-1 | Cluster 2 | 1 | 1 | 13 | 3 | 17 | 22 | 13 | 3709 |
| **NM174** | USA | 1998 | This study | 11 | 11 | 5 | 2 | F1-1 | Cluster 2 | 1 | UA | 13 | 5 | 17 | 160 | 13 | 1352 |
| **NM3147** | USA | 2009 | This study | 11 | 11 | 5 | 2 | F1-1 | Cluster 2 | 1 | 75 | 13 | 5 | 17 | 160 | 13 | 2055 |
| **NM3686** | Brazil | 1997 | This study | 11 | 11 | 5 | 2 | F1-1 | Cluster 2 | 1 | UA | 13 | 5 | 17 | UA | 31 | 2125 |
| **2001068** | Chad | 2001 | This study | 11 | 11 | 5 | 2 | F1-1 | Cluster 1 (Hajj cluster) | 1 | 1 | 13 | 5 | 72 | 9 | 31 | 68 |
| **2005079** | Chad | 2005 | This study | 11 | 11 | 5 | 2 | F1-1 | Cluster 1 (Hajj cluster) | 1 | 1 | 13 | 5 | 72 | 9 | 31 | 406 |
| **2001072** | Mauritius | 2001 | This study | 11 | 11 | 5 | 2 | F1-1 | Cluster 1 (Hajj cluster) | 1 | 1 | 13 | 5 | 72 | 9 | 31 | 4 |
| **2001073** | Mauritius | 2001 | This study | 11 | 11 | 5 | 2 | F1-1 | Cluster 1 (Hajj cluster) | 1 | 1 | 13 | 5 | 72 | 9 | 31 | 3 |
| **NM3684** | Netherlands | 1985 | This study | 1287 | 11 | 5 | 2 | F1-1 | Cluster 2 | 1 | 1 | 13 | 3 | 17 | 22 | 31 | 3856 |
| **2000081** | Senegal | 2000 | This study | 11 | 11 | 5 | 2 | F1-1 | Cluster 1 (Hajj cluster) | 1 | 1 | 13 | 5 | 72 | 9 | 31 | 199 |
| **M7089** | USA | 2000 | This study | 11 | 11 | 5 | 2 | F1-1 | Cluster 1 (Hajj cluster) | 1 | 1 | 13 | 5 | 72 | 9 | 31 | 3 |
| **2002004** | France | 2002 | This study | 11 | 11 | 5 | 2 | F1-1 | Cluster 2 | UA | UA | 13 | 5 | 17 | 160 | 575 | 1259 |
| **2004264** | Burkina Faso | 2004 | This study | 11 | 11 | 5 | 2 | F1-1 | Cluster 1 (Hajj cluster) | 1 | 1 | 13 | 5 | 72 | 9 | 576 | 192 |
| **2005040** | Chad | 2005 | This study | 11 | 11 | 5 | 2 | F1-1 | Cluster 1 (Hajj cluster) | 1 | 1 | 13 | 5 | 72 | 9 | 577 | 187 |
| **M12611** | Chile | 2011 | This study | 11 | 11 | 5 | 2 | F1-1 | Cluster 2 | 1 | 244 | 13 | 5 | 17 | 22 | N.D. | 1408 |
| **M1412** | Chile | 2012 | This study | 11 | 11 | 5 | 2 | F1-1 | Cluster 2 | 1 | 244 | 13 | 5 | 17 | 22 | N.D. | 1563 |
| **M9261** | Burkina Faso | 2002 | *Neisseria* Base^8^ | 11 | 11 | 5 | 2 | N.D. | Cluster 2 | 392 | 232 | 13 | 5 | 17 | 62 | 13 | 1260 |
| **M18774** | USA | 2008 | *Neisseria* Base | 11 | 11 | 5 | 2 | F1-94 | Cluster 2 | 141 | 244 | 309 | 5 | 17 | 22 | N.D. | N.D. |
| **27087** | Burkina Faso | 2002 | PubMLST^9^ | 11 | 11 | 5 | 2 | F1-1 | Cluster 2 | 1 | 232 | 13 | 5 | 17 | 62 | N.D. | N.D. |
| **26914** | Ireland | 2013 | PubMLST | 11 | 11 | 5 | 2 | F1-1 | Cluster 2 | 1 | 244 | 13 | 5 | 17 | 22 | N.D. | N.D. |
| **26898** | Ireland | 2013 | PubMLST | 11 | 11 | 5 | 2 | F1-1 | Cluster 2 | 1 | 244 | 13 | 5 | 17 | 22 | N.D. | N.D. |
| **26899** | Ireland | 2013 | PubMLST | 11 | 11 | 5 | 2 | F1-1 | Cluster 2 | 1 | 244 | 13 | 5 | 17 | 22 | N.D. | N.D. |
| **29333** | South Africa | 2003 | PubMLST | 11 | 11 | 5 | 2 | F1-1 | Cluster 1 (Hajj cluster) | 1 | 1 | 13 | 5 | 72 | 9 | N.D. | N.D. |
| **29334** | South Africa | 2003 | PubMLST | 11 | 11 | 5-1 | 2 | F1-1 | Cluster 2 | 324 | 1 | 13 | 3 | 17 | 22 | N.D. | N.D. |
| **29341** | South Africa | 2003 | PubMLST | 11 | 11 | 5-1 | 2 | F1-1 | Cluster 2 | 324 | 1 | 13 | 3 | 17 | 22 | N.D. | N.D. |
| **21587** | South Africa | 2003 | PubMLST | 11 | 11 | 5 | 2 | F1-1 | Cluster 2 | 1 | 311 | 13 | 5 | 17 | 160 | N.D. | N.D. |
| **29439** | South Africa | 2003 | PubMLST | 11 | 11 | 5 | 2 | F1-1 | Cluster 2 | 1 | 311 | 13 | 5 | 17 | 160 | N.D. | N.D. |
| **29324** | South Africa | 2003 | PubMLST | 11 | 11 | 5 | 2 | F1-1 | Cluster 2 | 1 | 1 | 695 | 3 | 17 | 22 | N.D. | N.D. |
| **29325** | South Africa | 2003 | PubMLST | 11 | 11 | 5 | 2 | F3-20 | Cluster 2 | 1 | 1 | 722 | 3 | 17 | 22 | N.D. | N.D. |
| **21578** | South Africa | 2004 | PubMLST | 11 | 11 | 5 | 2 | F1-1 | Cluster 1 (Hajj cluster) | 1 | 1 | 13 | 5 | 72 | 9 | N.D. | N.D. |
| **29316** | South Africa | 2004 | PubMLST | 11 | 11 | 5 | 2 | F1-1 | Cluster 1 (Hajj cluster) | 1 | 1 | 13 | 5 | 72 | 9 | N.D. | N.D. |
| **29330** | South Africa | 2004 | PubMLST | 11 | 11 | 5 | 2 | F1-1 | Cluster 2 | 1 | 311 | 13 | 5 | 17 | 160 | N.D. | N.D. |
| **29315** | South Africa | 2004 | PubMLST | 11 | 11 | 5 | 2 | F1-1 | Cluster 2 | 1 | 311 | 13 | 5 | 17 | 160 | N.D. | N.D. |
| **29329** | South Africa | 2004 | PubMLST | 11 | 11 | 5 | 2 | F1-1 | Cluster 2 | 1 | 311 | 13 | 5 | 17 | 160 | N.D. | N.D. |
| **29381** | South Africa | 2004 | PubMLST | 11 | 11 | 5 | 2 | F1-1 | Cluster 2 | 1 | 311 | 13 | 5 | 17 | 160 | N.D. | N.D. |
| **29411** | South Africa | 2004 | PubMLST | 11 | 11 | 5 | 2 | F1-1 | Cluster 2 | 1 | 311 | 13 | 5 | 17 | 160 | N.D. | N.D. |
| **29394** | South Africa | 2004 | PubMLST | 11 | 11 | 5-1 | 2 | F5-8 | Cluster 2 | 324 | 1 | 719 | 3 | 17 | 4 | N.D. | N.D. |
| **21588** | South Africa | 2005 | PubMLST | 11 | 11 | 5 | 2 | F1-1 | Cluster 1 (Hajj cluster) | 1 | 1 | 13 | 5 | 72 | 9 | N.D. | N.D. |
| **29326** | South Africa | 2005 | PubMLST | 11 | 11 | 5 | 2 | F1-1 | Cluster 1 (Hajj cluster) | 1 | 1 | 13 | 5 | 72 | 9 | N.D. | N.D. |
| **29337** | South Africa | 2005 | PubMLST | 11 | 11 | 5 | 2 | F1-1 | Cluster 1 (Hajj cluster) | 1 | 1 | 13 | 5 | 72 | 9 | N.D. | N.D. |
| **29370** | South Africa | 2005 | PubMLST | 11 | 11 | 5 | 2 | F1-1 | Cluster 1 (Hajj cluster) | 1 | 1 | 13 | 5 | 72 | 9 | N.D. | N.D. |
| **29371** | South Africa | 2005 | PubMLST | 11 | 11 | 5 | 2 | F1-1 | Cluster 1 (Hajj cluster) | 1 | 1 | 13 | 5 | 72 | 9 | N.D. | N.D. |
| **29384** | South Africa | 2005 | PubMLST | 11 | 11 | 5 | 2 | F1-1 | Cluster 1 (Hajj cluster) | 1 | 1 | 13 | 5 | 72 | 9 | N.D. | N.D. |
| **29401** | South Africa | 2005 | PubMLST | 11 | 11 | 5 | 2 | F1-1 | Cluster 1 (Hajj cluster) | 1 | 1 | 13 | 5 | 72 | 9 | N.D. | N.D. |
| **29422** | South Africa | 2005 | PubMLST | 11 | 11 | 5 | 2 | F1-1 | Cluster 1 (Hajj cluster) | 1 | 1 | 13 | 5 | 72 | 9 | N.D. | N.D. |
| **29331** | South Africa | 2005 | PubMLST | 11 | 11 | 5 | 2 | F1-1 | Cluster 2 | 1 | 1 | 695 | 3 | 17 | 22 | N.D. | N.D. |
| **21584** | South Africa | 2006 | PubMLST | 11 | 11 | 5 | 2 | F1-1 | Cluster 1 (Hajj cluster) | 1 | 1 | 13 | 5 | 72 | 9 | N.D. | N.D. |
| **29320** | South Africa | 2006 | PubMLST | 11 | 11 | 5 | 2 | F1-1 | Cluster 1 (Hajj cluster) | 1 | 1 | 13 | 5 | 72 | 9 | N.D. | N.D. |
| **29338** | South Africa | 2006 | PubMLST | 11 | 11 | 5 | 2 | F1-1 | Cluster 1 (Hajj cluster) | 1 | 1 | 13 | 5 | 72 | 9 | N.D. | N.D. |
| **29351** | South Africa | 2006 | PubMLST | 11 | 11 | 5 | 2 | F1-1 | Cluster 1 (Hajj cluster) | 1 | 1 | 13 | 5 | 72 | 9 | N.D. | N.D. |
| **29374** | South Africa | 2006 | PubMLST | 11 | 11 | 5 | 2 | F1-1 | Cluster 1 (Hajj cluster) | 1 | 1 | 13 | 5 | 72 | 9 | N.D. | N.D. |
| **29397** | South Africa | 2006 | PubMLST | 11 | 11 | 5 | 2 | F1-1 | Cluster 1 (Hajj cluster) | 1 | 1 | 13 | 5 | 72 | 9 | N.D. | N.D. |
| **29421** | South Africa | 2006 | PubMLST | 11 | 11 | 5 | 2 | F1-1 | Cluster 1 (Hajj cluster) | 1 | 1 | 13 | 5 | 72 | 9 | N.D. | N.D. |
| **29437** | South Africa | 2006 | PubMLST | 11 | 11 | 5 | 2 | F1-1 | Cluster 1 (Hajj cluster) | 1 | 1 | 13 | 5 | 72 | 9 | N.D. | N.D. |
| **29426** | South Africa | 2006 | PubMLST | 11 | 11 | 5 | 2 | F1-1 | Cluster 2 | 1 | 1 | 695 | 3 | 17 | 22 | N.D. | N.D. |
| **29322** | South Africa | 2007 | PubMLST | 11 | 11 | 5 | 2 | F1-1 | Cluster 1 (Hajj cluster) | 1 | 1 | 13 | 5 | 72 | 9 | N.D. | N.D. |
| **29342** | South Africa | 2007 | PubMLST | 11 | 11 | 5 | 2 | F1-1 | Cluster 1 (Hajj cluster) | 1 | 1 | 13 | 5 | 72 | 9 | N.D. | N.D. |
| **29357** | South Africa | 2007 | PubMLST | 11 | 11 | 5 | 2 | F1-1 | Cluster 1 (Hajj cluster) | 1 | 1 | 13 | 5 | 72 | 9 | N.D. | N.D. |
| **29377** | South Africa | 2007 | PubMLST | 11 | 11 | 5 | 2 | F1-1 | Cluster 1 (Hajj cluster) | 1 | 1 | 13 | 5 | 72 | 9 | N.D. | N.D. |
| **29419** | South Africa | 2007 | PubMLST | 11 | 11 | 5 | 2 | F1-1 | Cluster 1 (Hajj cluster) | 1 | 1 | 13 | 5 | 72 | 9 | N.D. | N.D. |
| **29424** | South Africa | 2007 | PubMLST | 11 | 11 | 5 | 2 | F1-1 | Cluster 1 (Hajj cluster) | 1 | 1 | 13 | 5 | 72 | 9 | N.D. | N.D. |
| **29438** | South Africa | 2007 | PubMLST | 4977 | 11 | 5 | 2 | F1-1 | Cluster 1 (Hajj cluster) | 1 | 1 | 13 | 5 | 72 | 9 | N.D. | N.D. |
| **29375** | South Africa | 2007 | PubMLST | 11 | 11 | 5 | 2 | F1-1 | Cluster 1 (Hajj cluster) | 1 | 1 | 725 | 5 | 72 | 9 | N.D. | N.D. |
| **21581** | South Africa | 2007 | PubMLST | 11 | 11 | 5-1 | 2 | F1-1 | Cluster 2 | 324 | 1 | 13 | 3 | 17 | 22 | N.D. | N.D. |
| **29409** | South Africa | 2007 | PubMLST | 11 | 11 | 5 | 2 | F1-1 | Cluster 2 | 1 | 1 | 695 | 3 | 17 | 22 | N.D. | N.D. |
| **21573** | South Africa | 2008 | PubMLST | 11 | 11 | 5 | 2 | F1-1 | Cluster 1 (Hajj cluster) | 1 | 1 | 13 | 5 | 72 | 9 | N.D. | N.D. |
| **29317** | South Africa | 2008 | PubMLST | 11 | 11 | 5 | 2 | F1-1 | Cluster 1 (Hajj cluster) | 1 | 1 | 13 | 5 | 72 | 9 | N.D. | N.D. |
| **29359** | South Africa | 2008 | PubMLST | 11 | 11 | 5 | 2 | F1-1 | Cluster 1 (Hajj cluster) | 1 | 1 | 13 | 5 | 72 | 9 | N.D. | N.D. |
| **29383** | South Africa | 2008 | PubMLST | 11 | 11 | 5 | 2 | F1-1 | Cluster 1 (Hajj cluster) | 1 | 1 | 13 | 5 | 72 | 9 | N.D. | N.D. |
| **29393** | South Africa | 2008 | PubMLST | 11 | 11 | 5 | 2 | F1-1 | Cluster 1 (Hajj cluster) | 1 | 1 | 13 | 5 | 72 | 9 | N.D. | N.D. |
| **29395** | South Africa | 2008 | PubMLST | 11 | 11 | 5 | 2 | F1-1 | Cluster 1 (Hajj cluster) | 1 | 1 | 13 | 5 | 72 | 9 | N.D. | N.D. |
| **29410** | South Africa | 2008 | PubMLST | 11 | 11 | 5 | 2 | F1-1 | Cluster 1 (Hajj cluster) | 1 | 1 | 13 | 5 | 72 | 9 | N.D. | N.D. |
| **29415** | South Africa | 2008 | PubMLST | 11 | 11 | 5 | 2 | F1-1 | Cluster 1 (Hajj cluster) | 1 | 1 | 13 | 5 | 72 | 9 | N.D. | N.D. |
| **29416** | South Africa | 2008 | PubMLST | 11 | 11 | 5 | 2 | F1-1 | Cluster 1 (Hajj cluster) | 1 | 1 | 13 | 5 | 72 | 9 | N.D. | N.D. |
| **29340** | South Africa | 2008 | PubMLST | 11 | 11 | 5-1 | 2 | F5-8 | Cluster 2 | 324 | 1 | 719 | 3 | 17 | 4 | N.D. | N.D. |
| **29441** | South Africa | 2008 | PubMLST | 11 | 11 | 5-1 | 2 | F5-8 | Cluster 2 | 324 | 1 | 719 | 3 | 17 | 4 | N.D. | N.D. |
| **29347** | South Africa | 2009 | PubMLST | 11 | 11 | 5 | 2 | F1-1 | Cluster 1 (Hajj cluster) | 1 | 1 | 13 | 5 | 72 | 9 | N.D. | N.D. |
| **29355** | South Africa | 2009 | PubMLST | 11 | 11 | 5 | 2 | F1-1 | Cluster 1 (Hajj cluster) | 1 | 1 | 13 | 5 | 72 | 9 | N.D. | N.D. |
| **29356** | South Africa | 2009 | PubMLST | 11 | 11 | 5 | 2 | F1-1 | Cluster 1 (Hajj cluster) | 1 | 1 | 13 | 5 | 72 | 9 | N.D. | N.D. |
| **29367** | South Africa | 2009 | PubMLST | 11 | 11 | 5 | 2 | F1-1 | Cluster 1 (Hajj cluster) | 1 | 1 | 13 | 5 | 72 | 9 | N.D. | N.D. |
| **29408** | South Africa | 2009 | PubMLST | 11 | 11 | 5 | 2 | F1-1 | Cluster 1 (Hajj cluster) | 1 | 1 | 13 | 5 | 72 | 9 | N.D. | N.D. |
| **29435** | South Africa | 2009 | PubMLST | 11 | 11 | 5 | 2 | F1-1 | Cluster 1 (Hajj cluster) | 1 | 1 | 13 | 5 | 72 | 9 | N.D. | N.D. |
| **29339** | South Africa | 2009 | PubMLST | 11 | 11 | 5 | 2 | F1-1 | Cluster 1 (Hajj cluster) | 1 | 1 | 695 | 5 | 72 | 9 | N.D. | N.D. |
| **29348** | South Africa | 2009 | PubMLST | 11 | 11 | 5 | 2 | F1-1 | Cluster 1 (Hajj cluster) | 1 | 1 | 724 | 5 | 72 | 9 | N.D. | N.D. |
| **29376** | South Africa | 2009 | PubMLST | 11 | 11 | 5 | 2 | F1-1 | Cluster 2 | 1 | 1 | 695 | 3 | 17 | 22 | N.D. | N.D. |
| **29399** | South Africa | 2009 | PubMLST | 11 | 11 | 5 | 2 | F1-1 | Cluster 2 | 1 | 1 | 695 | 3 | 17 | 22 | N.D. | N.D. |
| **29385** | South Africa | 2009 | PubMLST | 11 | 11 | 5-1 | 2 | F5-8 | Cluster 2 | 324 | 1 | 719 | 3 | 17 | 4 | N.D. | N.D. |
| **21582** | South Africa | 2010 | PubMLST | 11 | 11 | 5 | 2 | F1-1 | Cluster 1 (Hajj cluster) | 1 | 1 | 13 | 5 | 72 | 9 | N.D. | N.D. |
| **29332** | South Africa | 2010 | PubMLST | 11 | 11 | 5 | 2 | F1-1 | Cluster 1 (Hajj cluster) | 1 | 1 | 13 | 5 | 72 | 9 | N.D. | N.D. |
| **29336** | South Africa | 2010 | PubMLST | 11 | 11 | 5 | 2 | F1-1 | Cluster 1 (Hajj cluster) | 1 | 1 | 13 | 5 | 72 | 9 | N.D. | N.D. |
| **29386** | South Africa | 2010 | PubMLST | 11 | 11 | 5 | 2 | F1-1 | Cluster 1 (Hajj cluster) | 1 | 1 | 13 | 5 | 72 | 9 | N.D. | N.D. |
| **29388** | South Africa | 2010 | PubMLST | 11 | 11 | 5 | 2 | F1-1 | Cluster 1 (Hajj cluster) | 1 | 1 | 13 | 5 | 72 | 9 | N.D. | N.D. |
| **29412** | South Africa | 2010 | PubMLST | 11 | 11 | 5 | 2 | F1-1 | Cluster 1 (Hajj cluster) | 1 | 1 | 13 | 5 | 72 | 9 | N.D. | N.D. |
| **29414** | South Africa | 2010 | PubMLST | 1287 | 11 | 5 | 2 | F1-1 | Cluster 1 (Hajj cluster) | 1 | 1 | 13 | 5 | 72 | 9 | N.D. | N.D. |
| **29425** | South Africa | 2010 | PubMLST | 11 | 11 | 5 | 2 | F1-1 | Cluster 1 (Hajj cluster) | 1 | 1 | 13 | 5 | 72 | 9 | N.D. | N.D. |
| **29417** | South Africa | 2010 | PubMLST | 11 | 11 | 5 | 2 | F1-6 | Cluster 1 (Hajj cluster) | 1 | 1 | 730 | 5 | 72 | 9 | N.D. | N.D. |
| **29328** | South Africa | 2010 | PubMLST | 11 | 11 | 5 | 2 | F1-1 | Cluster 2 | 1 | 1 | 695 | 3 | 17 | 22 | N.D. | N.D. |
| **29349** | South Africa | 2010 | PubMLST | 11 | 11 | 5 | 2 | F1-1 | Cluster 2 | 1 | 1 | 695 | 3 | 17 | 22 | N.D. | N.D. |
| **21583** | South Africa | 2011 | PubMLST | 11 | 11 | 5 | 2 | F1-1 | Cluster 1 (Hajj cluster) | 1 | 1 | 13 | 5 | 72 | 9 | N.D. | N.D. |
| **29361** | South Africa | 2011 | PubMLST | 11 | 11 | 5 | 2 | F1-1 | Cluster 1 (Hajj cluster) | 1 | 1 | 13 | 5 | 72 | 9 | N.D. | N.D. |
| **29368** | South Africa | 2011 | PubMLST | 11 | 11 | 5 | 2 | F1-1 | Cluster 1 (Hajj cluster) | 1 | 1 | 13 | 5 | 72 | 9 | N.D. | N.D. |
| **29418** | South Africa | 2011 | PubMLST | 11 | 11 | 5 | 2 | F1-1 | Cluster 1 (Hajj cluster) | 1 | 1 | 13 | 5 | 72 | 9 | N.D. | N.D. |
| **29428** | South Africa | 2011 | PubMLST | 11 | 11 | 5 | 2 | F1-1 | Cluster 1 (Hajj cluster) | 1 | 1 | 13 | 5 | 72 | 9 | N.D. | N.D. |
| **29400** | South Africa | 2011 | PubMLST | 11 | 11 | 5 | 2 | F1-1 | Cluster 1 (Hajj cluster) | 1 | 1 | 729 | 5 | 72 | 9 | N.D. | N.D. |
| **29364** | South Africa | 2011 | PubMLST | 11 | 11 | 5 | 2 | F1-1 | Cluster 2 | 1 | 1 | 695 | 3 | 17 | 22 | N.D. | N.D. |
| **29407** | South Africa | 2011 | PubMLST | 11 | 11 | 5 | 2 | F1-1 | Cluster 2 | 1 | 1 | 695 | 3 | 17 | 22 | N.D. | N.D. |
| **29420** | South Africa | 2011 | PubMLST | 11 | 11 | 5-1 | 2 | F5-8 | Cluster 2 | 324 | 1 | 719 | 3 | 17 | 4 | N.D. | N.D. |
| **29360** | South Africa | 2012 | PubMLST | 11 | 11 | 5 | 2 | F1-1 | Cluster 1 (Hajj cluster) | 1 | 1 | 13 | 5 | 72 | 9 | N.D. | N.D. |
| **29346** | South Africa | 2012 | PubMLST | 11 | 11 | 5 | 2 | F1-1 | Cluster 1 (Hajj cluster) | 1 | 1 | 13 | 5 | 72 | 9 | N.D. | N.D. |
| **29369** | South Africa | 2012 | PubMLST | 11 | 11 | 5 | 2 | F1-1 | Cluster 1 (Hajj cluster) | 1 | 1 | 13 | 5 | 72 | 9 | N.D. | N.D. |
| **29389** | South Africa | 2012 | PubMLST | 11 | 11 | 5 | 2 | F1-1 | Cluster 1 (Hajj cluster) | 1 | 1 | 13 | 5 | 72 | 9 | N.D. | N.D. |
| **29405** | South Africa | 2012 | PubMLST | 11 | 11 | 5 | 2 | F1-1 | Cluster 1 (Hajj cluster) | 1 | 1 | 13 | 5 | 72 | 9 | N.D. | N.D. |
| **29373** | South Africa | 2012 | PubMLST | 11 | 11 | 5 | 2 | F1-94 | Cluster 1 (Hajj cluster) | 1 | 1 | 309 | 5 | 72 | 9 | N.D. | N.D. |
| **29372** | South Africa | 2012 | PubMLST | 11 | 11 | 5 | 2 | F1-1 | Cluster 2 | 1 | 1 | 695 | 3 | 17 | 22 | N.D. | N.D. |
| **29402** | South Africa | 2013 | PubMLST | 11 | 11 | 5 | 2 | F1-1 | Cluster 1 (Hajj cluster) | 1 | 1 | 13 | 5 | 72 | 9 | N.D. | N.D. |
| **29387** | South Africa | 2013 | PubMLST | 11 | 11 | 5 | 2 | F1-1 | Cluster 1 (Hajj cluster) | 1 | 1 | 13 | 5 | 72 | 9 | N.D. | N.D. |
| **29313** | South Africa | 2013 | PubMLST | 11 | 11 | 5 | 2 | F3-27 | Cluster 1 (Hajj cluster) | 1 | 1 | 321 | 5 | 72 | 9 | N.D. | N.D. |
| **29318** | South Africa | 2013 | PubMLST | 11 | 11 | 5 | 2 | F3-27 | Cluster 1 (Hajj cluster) | 1 | 1 | 321 | 5 | 72 | 9 | N.D. | N.D. |
| **29314** | South Africa | 2013 | PubMLST | 11 | 11 | 5 | 2 | F1-1 | Cluster 1 (Hajj cluster) | 1 | 1 | 695 | 5 | 72 | 9 | N.D. | N.D. |
| **29423** | South Africa | 2013 | PubMLST | 11 | 11 | 5 | 2 | F1-1 | Cluster 2 | 1 | 1 | 731 | 3 | 17 | 22 | N.D. | N.D. |
| **29649** | UK | 1975 | PubMLST | 11 | 11 | 5 | 2 | F1-1 | Cluster 2 | 1 | 1 | 13 | 3 | 17 | 22 | N.D. | N.D. |
| **29648** | UK | 1975 | PubMLST | 11 | 11 | 5 | 2 | F1-1 | Cluster 2 | 1 | 1 | 13 | 3 | 17 | 22 | N.D. | N.D. |
| **29650** | UK | 1975 | PubMLST | 473 | 11 | 5 | 2 | F1-1 | Cluster 2 | 1 | 1 | 13 | 3 | 17 | 22 | N.D. | N.D. |
| **29651** | UK | 1975 | PubMLST | 11 | 11 | 5 | 2 | F1-1 | Cluster 2 | 1 | 1 | 13 | 3 | 17 | 22 | N.D. | N.D. |
| **29652** | UK | 1975 | PubMLST | 473 | 11 | 5 | 2 | F1-1 | Cluster 2 | 1 | 1 | 13 | 3 | 17 | 22 | N.D. | N.D. |
| **19957** | UK | 2000 | PubMLST | 11 | 11 | 5 | 2 | F1-1 | Cluster 1 (Hajj cluster) | 1 | 1 | 13 | 5 | 72 | 9 | N.D. | N.D. |
| **29775** | UK | 2000 | PubMLST | 11 | 11 | 5 | 2 | F1-1 | Cluster 1 (Hajj cluster) | 1 | 1 | 13 | 5 | 72 | 9 | N.D. | N.D. |
| **29776** | UK | 2000 | PubMLST | 11 | 11 | 5 | 2 | F1-1 | Cluster 1 (Hajj cluster) | 1 | 1 | 13 | 5 | 72 | 9 | N.D. | N.D. |
| **29778** | UK | 2000 | PubMLST | 11 | 11 | 5 | 2 | F1-1 | Cluster 1 (Hajj cluster) | 1 | 1 | 13 | 5 | 72 | 9 | N.D. | N.D. |
| **29928** | UK | 2000 | PubMLST | 11 | 11 | 5 | 2 | F1-1 | Cluster 1 (Hajj cluster) | 1 | 1 | 13 | 5 | 72 | 9 | N.D. | N.D. |
| **29929** | UK | 2000 | PubMLST | 11 | 11 | 5 | 2 | F1-1 | Cluster 1 (Hajj cluster) | 1 | 1 | 13 | 5 | 72 | 9 | N.D. | N.D. |
| **29930** | UK | 2000 | PubMLST | 11 | 11 | 5 | 2 | F1-1 | Cluster 1 (Hajj cluster) | 1 | 1 | 13 | 5 | 72 | 9 | N.D. | N.D. |
| **29931** | UK | 2000 | PubMLST | 11 | 11 | 5 | 2 | F1-1 | Cluster 1 (Hajj cluster) | 1 | 1 | 13 | 5 | 72 | 9 | N.D. | N.D. |
| **29932** | UK | 2000 | PubMLST | 11 | 11 | 5 | 2 | F1-1 | Cluster 1 (Hajj cluster) | 1 | 1 | 13 | 5 | 72 | 9 | N.D. | N.D. |
| **29933** | UK | 2000 | PubMLST | 11 | 11 | 5 | 2 | F1-1 | Cluster 1 (Hajj cluster) | 1 | 1 | 13 | 5 | 72 | 9 | N.D. | N.D. |
| **29934** | UK | 2000 | PubMLST | 11 | 11 | 5 | 2 | F1-1 | Cluster 1 (Hajj cluster) | 1 | 1 | 13 | 5 | 72 | 9 | N.D. | N.D. |
| **29935** | UK | 2000 | PubMLST | 11 | 11 | 5 | 2 | F1-1 | Cluster 1 (Hajj cluster) | 1 | 1 | 13 | 5 | 72 | 9 | N.D. | N.D. |
| **29936** | UK | 2000 | PubMLST | 11 | 11 | 5 | 2 | F1-1 | Cluster 1 (Hajj cluster) | 1 | 1 | 13 | 5 | 72 | 9 | N.D. | N.D. |
| **29937** | UK | 2000 | PubMLST | 11 | 11 | 5 | 2 | F1-1 | Cluster 1 (Hajj cluster) | 1 | 1 | 13 | 5 | 72 | 9 | N.D. | N.D. |
| **29938** | UK | 2000 | PubMLST | 11 | 11 | 5 | 2 | F1-1 | Cluster 1 (Hajj cluster) | 1 | 1 | 13 | 5 | 72 | 9 | N.D. | N.D. |
| **29939** | UK | 2000 | PubMLST | 11 | 11 | 5 | 2 | F1-1 | Cluster 1 (Hajj cluster) | 1 | 1 | 13 | 5 | 72 | 9 | N.D. | N.D. |
| **29940** | UK | 2000 | PubMLST | 11 | 11 | 5 | 2 | F1-1 | Cluster 1 (Hajj cluster) | 1 | 1 | 13 | 5 | 72 | 9 | N.D. | N.D. |
| **29941** | UK | 2000 | PubMLST | 11 | 11 | 5 | 2 | F1-1 | Cluster 1 (Hajj cluster) | 1 | 1 | 13 | 5 | 72 | 9 | N.D. | N.D. |
| **29942** | UK | 2000 | PubMLST | 11 | 11 | 5 | 2 | F1-1 | Cluster 1 (Hajj cluster) | 1 | 1 | 13 | 5 | 72 | 9 | N.D. | N.D. |
| **29943** | UK | 2000 | PubMLST | 11 | 11 | 5 | 2 | F1-1 | Cluster 1 (Hajj cluster) | 1 | 1 | 13 | 5 | 72 | 9 | N.D. | N.D. |
| **29944** | UK | 2000 | PubMLST | 11 | 11 | 5 | 2 | F1-1 | Cluster 1 (Hajj cluster) | 1 | 1 | 13 | 5 | 72 | 9 | N.D. | N.D. |
| **29945** | UK | 2000 | PubMLST | 11 | 11 | 5 | 2 | F1-1 | Cluster 1 (Hajj cluster) | 1 | 1 | 13 | 5 | 72 | 9 | N.D. | N.D. |
| **29946** | UK | 2000 | PubMLST | 11 | 11 | 5 | 2 | F1-1 | Cluster 1 (Hajj cluster) | 1 | 1 | 13 | 5 | 72 | 9 | N.D. | N.D. |
| **29947** | UK | 2000 | PubMLST | 11 | 11 | 5 | 2 | F1-1 | Cluster 1 (Hajj cluster) | 1 | 1 | 13 | 5 | 72 | 9 | N.D. | N.D. |
| **29948** | UK | 2000 | PubMLST | 11 | 11 | 5 | 2 | F1-1 | Cluster 1 (Hajj cluster) | 1 | 1 | 13 | 5 | 72 | 9 | N.D. | N.D. |
| **29949** | UK | 2000 | PubMLST | 11 | 11 | 5 | 2 | F1-1 | Cluster 1 (Hajj cluster) | 1 | 1 | 13 | 5 | 72 | 9 | N.D. | N.D. |
| **29950** | UK | 2000 | PubMLST | 11 | 11 | 5 | 2 | F1-1 | Cluster 1 (Hajj cluster) | 1 | 1 | 13 | 5 | 72 | 9 | N.D. | N.D. |
| **29681** | UK | 2001 | PubMLST | 11 | 11 | 5 | 2 | F1-1 | Cluster 1 (Hajj cluster) | 1 | 1 | 13 | 5 | 72 | 9 | N.D. | N.D. |
| **29677** | UK | 2001 | PubMLST | 11 | 11 | 5 | 2 | F1-1 | Cluster 1 (Hajj cluster) | 1 | 1 | 13 | 5 | 72 | 9 | N.D. | N.D. |
| **29680** | UK | 2001 | PubMLST | 11 | 11 | 5 | 2 | F1-1 | Cluster 1 (Hajj cluster) | 1 | 1 | 13 | 5 | 72 | 9 | N.D. | N.D. |
| **29684** | UK | 2001 | PubMLST | 11 | 11 | 5 | 2 | F1-1 | Cluster 1 (Hajj cluster) | 1 | 1 | 13 | 5 | 72 | 9 | N.D. | N.D. |
| **29679** | UK | 2001 | PubMLST | 11 | 11 | 5 | 2 | F1-1 | Cluster 2 | 1 | 1 | 13 | 5 | 72 | 22 | N.D. | N.D. |
| **29683** | UK | 2002 | PubMLST | 11 | 11 | 5 | 2 | F1-1 | Cluster 1 (Hajj cluster) | 1 | 1 | 13 | 5 | 72 | 9 | N.D. | N.D. |
| **29686** | UK | 2002 | PubMLST | 11 | 11 | 5 | 2 | F1-1 | Cluster 1 (Hajj cluster) | 1 | 1 | 13 | 5 | 72 | 9 | N.D. | N.D. |
| **29688** | UK | 2002 | PubMLST | 11 | 11 | 5 | 2 | F1-1 | Cluster 1 (Hajj cluster) | 1 | 1 | 13 | 5 | 72 | 9 | N.D. | N.D. |
| **29689** | UK | 2002 | PubMLST | 11 | 11 | 5 | 2 | F1-1 | Cluster 1 (Hajj cluster) | 1 | 1 | 13 | 5 | 72 | 9 | N.D. | N.D. |
| **29690** | UK | 2002 | PubMLST | 11 | 11 | 5 | 2 | F1-1 | Cluster 1 (Hajj cluster) | 1 | 1 | 13 | 5 | 72 | 9 | N.D. | N.D. |
| **29692** | UK | 2002 | PubMLST | 11 | 11 | 5 | 2 | F1-1 | Cluster 1 (Hajj cluster) | 1 | 1 | 13 | 5 | 72 | 9 | N.D. | N.D. |
| **29694** | UK | 2002 | PubMLST | 11 | 11 | 5 | 2 | F1-1 | Cluster 1 (Hajj cluster) | 1 | 1 | 13 | 5 | 72 | 9 | N.D. | N.D. |
| **29695** | UK | 2002 | PubMLST | 11 | 11 | 5 | 2 | F1-1 | Cluster 1 (Hajj cluster) | 1 | 1 | 13 | 5 | 72 | 9 | N.D. | N.D. |
| **29696** | UK | 2002 | PubMLST | 11 | 11 | 5 | 2 | F1-1 | Cluster 1 (Hajj cluster) | 1 | 1 | 13 | 5 | 72 | 9 | N.D. | N.D. |
| **29697** | UK | 2002 | PubMLST | 11 | 11 | 5 | 2 | F1-1 | Cluster 1 (Hajj cluster) | 1 | 1 | 13 | 5 | 72 | 9 | N.D. | N.D. |
| **29700** | UK | 2003 | PubMLST | 11 | 11 | 5 | 2 | F1-1 | Cluster 1 (Hajj cluster) | 1 | 1 | 13 | 5 | 72 | 9 | N.D. | N.D. |
| **29702** | UK | 2004 | PubMLST | 11 | 11 | 5 | 2 | F1-1 | Cluster 1 (Hajj cluster) | 1 | 1 | 13 | 5 | 72 | 9 | N.D. | N.D. |
| **29703** | UK | 2004 | PubMLST | 11 | 11 | 5 | 2 | F1-1 | Cluster 1 (Hajj cluster) | 1 | 1 | 13 | 5 | 72 | 9 | N.D. | N.D. |
| **29705** | UK | 2006 | PubMLST | 11 | 11 | 5 | 2 | F1-1 | Cluster 2 | 1 | 1 | 13 | 5 | 72 | 22 | N.D. | N.D. |
| **29707** | UK | 2007 | PubMLST | 11 | 11 | 5 | 2 | F1-1 | Cluster 1 (Hajj cluster) | 1 | 1 | 13 | 5 | 72 | 9 | N.D. | N.D. |
| **29710** | UK | 2007 | PubMLST | 247 | 11 | 5-2 | 10 | F3-1 | Cluster 2 | 8 | 1 | 421 | 3 | 17 | 36 | N.D. | N.D. |
| **29715** | UK | 2009 | PubMLST | 11 | 11 | 5 | 2 | F1-1 | Cluster 2 | 1 | 244 | 13 | 5 | 17 | 22 | N.D. | N.D. |
| **29716** | UK | 2009 | PubMLST | 11 | 11 | 5 | 2 | F1-1 | Cluster 2 | 1 | 244 | 13 | 5 | 17 | 22 | N.D. | N.D. |
| **20057** | UK | 2010 | PubMLST | 11 | 11 | 5 | 2 | F1-1 | Cluster 2 | 1 | 249 | 13 | 5 | 17 | 22 | N.D. | N.D. |
| **29714** | UK | 2010 | PubMLST | 11 | 11 | 5 | 2 | F1-1 | Cluster 2 | 1 | 455 | 13 | 5 | 17 | 22 | N.D. | N.D. |
| **19968** | UK | 2010 | PubMLST | 11 | 11 | 5 | 2 | F1-1 | Cluster 2 | 1 | 244 | 13 | 5 | 17 | 22 | N.D. | N.D. |
| **20154** | UK | 2010 | PubMLST | 11 | 11 | 5 | 2 | F1-1 | Cluster 2 | 1 | 244 | 13 | 5 | 17 | 22 | N.D. | N.D. |
| **20158** | UK | 2010 | PubMLST | 11 | 11 | 5 | 2 | F1-1 | Cluster 2 | 1 | 244 | 13 | 5 | 17 | 22 | N.D. | N.D. |
| **29718** | UK | 2010 | PubMLST | 11 | 11 | 5 | 2 | F1-1 | Cluster 2 | 1 | 244 | 13 | 5 | 17 | 22 | N.D. | N.D. |
| **29719** | UK | 2010 | PubMLST | 11 | 11 | 5 | 2 | F1-1 | Cluster 2 | 1 | 244 | 13 | 5 | 17 | 22 | N.D. | N.D. |
| **29720** | UK | 2010 | PubMLST | 11 | 11 | 5 | 2 | F1-1 | Cluster 2 | 1 | 244 | 13 | 5 | 17 | 22 | N.D. | N.D. |
| **20424** | UK | 2011 | PubMLST | 11 | 11 | 5 | 2 | F1-1 | Cluster 1 (Hajj cluster) | 1 | 1 | 13 | 5 | 72 | 9 | N.D. | N.D. |
| **20196** | UK | 2011 | PubMLST | 11 | 11 | 5 | 2 | F1-1 | Cluster 2 | 1 | 244 | 13 | 5 | 17 | 22 | N.D. | N.D. |
| **20216** | UK | 2011 | PubMLST | 11 | 11 | 5 | 2 | F1-1 | Cluster 2 | 1 | 244 | 13 | 5 | 17 | 22 | N.D. | N.D. |
| **20226** | UK | 2011 | PubMLST | 11 | 11 | 5 | 2 | F1-1 | Cluster 2 | 1 | 244 | 13 | 5 | 17 | 22 | N.D. | N.D. |
| **20247** | UK | 2011 | PubMLST | 11 | 11 | 5 | 2 | F1-1 | Cluster 2 | 1 | 244 | 13 | 5 | 17 | 22 | N.D. | N.D. |
| **20288** | UK | 2011 | PubMLST | 11 | 11 | 5 | 2 | F1-1 | Cluster 2 | 1 | 244 | 13 | 5 | 17 | 22 | N.D. | N.D. |
| **20368** | UK | 2011 | PubMLST | 11 | 11 | 5 | 2 | F1-1 | Cluster 2 | 1 | 244 | 13 | 5 | 17 | 22 | N.D. | N.D. |
| **20436** | UK | 2011 | PubMLST | 11 | 11 | 5 | 2 | F1-1 | Cluster 2 | 1 | 244 | 13 | 5 | 17 | 22 | N.D. | N.D. |
| **20449** | UK | 2011 | PubMLST | 11 | 11 | 5 | 2 | F1-1 | Cluster 2 | 1 | 244 | 13 | 5 | 17 | 22 | N.D. | N.D. |
| **21163** | UK | 2011 | PubMLST | 11 | 11 | 5 | 2 | F1-1 | Cluster 2 | 1 | 244 | 13 | 5 | 17 | 22 | N.D. | N.D. |
| **21203** | UK | 2011 | PubMLST | 11 | 11 | 5 | 2 | F1-1 | Cluster 2 | 1 | 244 | 13 | 5 | 17 | 22 | N.D. | N.D. |
| **21206** | UK | 2011 | PubMLST | 11 | 11 | 5 | 2 | F1-1 | Cluster 2 | 1 | 244 | 13 | 5 | 17 | 22 | N.D. | N.D. |
| **21216** | UK | 2011 | PubMLST | 11 | 11 | 5 | 2 | F1-1 | Cluster 2 | 1 | 244 | 13 | 5 | 17 | 22 | N.D. | N.D. |
| **21123** | UK | 2011 | PubMLST | 1860 | 11 | 5 | 2 | F1-1 | Cluster 2 | 4 | 244 | 13 | 5 | 17 | 22 | N.D. | N.D. |
| **28119** | UK | 2012 | PubMLST | 11 | 11 | 5 | 2 | F1-5 | Cluster 2 | 1 | 244 | 20 | 5 | 17 | 22 | N.D. | N.D. |
| **21375** | UK | 2012 | PubMLST | 11 | 11 | 5 | 2 | F1-146 | Cluster 2 | 1 | 244 | 488 | 5 | 17 | 22 | N.D. | N.D. |
| **21381** | UK | 2012 | PubMLST | 11 | 11 | 5 | 2 | F1-146 | Cluster 2 | 1 | 244 | 488 | 5 | 17 | 22 | N.D. | N.D. |
| **21446** | UK | 2012 | PubMLST | 11 | 11 | 5 | 2 | F1-146 | Cluster 2 | 1 | 244 | 488 | 5 | 17 | 22 | N.D. | N.D. |
| **21386** | UK | 2012 | PubMLST | 10284 | 11 | 5 | 2 | F1-1 | Cluster 2 | 1 | 444 | 13 | 5 | 17 | 22 | N.D. | N.D. |
| **21492** | UK | 2012 | PubMLST | 11 | 11 | 5 | 2 | F1-1 | Cluster 2 | 1 | 455 | 13 | 5 | 17 | 22 | N.D. | N.D. |
| **20460** | UK | 2012 | PubMLST | 11 | 11 | 5 | 2 | F1-1 | Cluster 2 | 1 | 244 | 13 | 5 | 17 | 22 | N.D. | N.D. |
| **20461** | UK | 2012 | PubMLST | 11 | 11 | 5 | 2 | F1-1 | Cluster 2 | 1 | 244 | 13 | 5 | 17 | 22 | N.D. | N.D. |
| **20462** | UK | 2012 | PubMLST | 11 | 11 | 5 | 2 | F1-1 | Cluster 2 | 1 | 244 | 13 | 5 | 17 | 22 | N.D. | N.D. |
| **21288** | UK | 2012 | PubMLST | 11 | 11 | 5 | 2 | F1-1 | Cluster 2 | 1 | 244 | 13 | 5 | 17 | 22 | N.D. | N.D. |
| **21298** | UK | 2012 | PubMLST | 11 | 11 | 5 | 2 | F1-1 | Cluster 2 | 1 | 244 | 13 | 5 | 17 | 22 | N.D. | N.D. |
| **21302** | UK | 2012 | PubMLST | 11 | 11 | 5 | 2 | F1-1 | Cluster 2 | 1 | 244 | 13 | 5 | 17 | 22 | N.D. | N.D. |
| **21305** | UK | 2012 | PubMLST | 11 | 11 | 5 | 2 | F1-1 | Cluster 2 | 1 | 244 | 13 | 5 | 17 | 22 | N.D. | N.D. |
| **21334** | UK | 2012 | PubMLST | 11 | 11 | 5 | 2 | F1-1 | Cluster 2 | 1 | 244 | 13 | 5 | 17 | 22 | N.D. | N.D. |
| **21354** | UK | 2012 | PubMLST | 11 | 11 | 5 | 2 | F1-1 | Cluster 2 | 1 | 244 | 13 | 5 | 17 | 22 | N.D. | N.D. |
| **21377** | UK | 2012 | PubMLST | 11 | 11 | 5 | 2 | F1-1 | Cluster 2 | 1 | 244 | 13 | 5 | 17 | 22 | N.D. | N.D. |
| **21499** | UK | 2012 | PubMLST | 11 | 11 | 5 | 2 | F1-1 | Cluster 2 | 1 | 244 | 13 | 5 | 17 | 22 | N.D. | N.D. |
| **28115** | UK | 2012 | PubMLST | 11 | 11 | 5 | 2 | F1-1 | Cluster 2 | 1 | 244 | 13 | 5 | 17 | 22 | N.D. | N.D. |
| **28116** | UK | 2012 | PubMLST | 11 | 11 | 5 | 2 | F1-1 | Cluster 2 | 1 | 244 | 13 | 5 | 17 | 22 | N.D. | N.D. |
| **28121** | UK | 2012 | PubMLST | 10651 | 11 | 5 | 2 | F1-1 | Cluster 2 | 1 | 244 | 13 | 5 | 17 | 22 | N.D. | N.D. |
| **28122** | UK | 2012 | PubMLST | 11 | 11 | 5 | 2 | F1-1 | Cluster 2 | 1 | 244 | 13 | 5 | 17 | 22 | N.D. | N.D. |
| **28125** | UK | 2012 | PubMLST | 11 | 11 | 5 | 2 | F1-1 | Cluster 2 | 1 | 244 | 13 | 5 | 17 | 22 | N.D. | N.D. |
| **28128** | UK | 2012 | PubMLST | 11 | 11 | 5 | 2 | F1-1 | Cluster 2 | 1 | 244 | 13 | 5 | 17 | 22 | N.D. | N.D. |
| **28131** | UK | 2012 | PubMLST | 11 | 11 | 5 | 2 | F1-1 | Cluster 2 | 1 | 244 | 13 | 5 | 17 | 22 | N.D. | N.D. |
| **28132** | UK | 2012 | PubMLST | 11 | 11 | 5 | 2 | F1-1 | Cluster 2 | 1 | 244 | 13 | 5 | 17 | 22 | N.D. | N.D. |
| **28114** | UK | 2012 | PubMLST | 11 | 11 | 5-1 | 10-4 | F1-1 | Cluster 2 | 142 | 244 | 13 | 5 | 17 | 22 | N.D. | N.D. |
| **28117** | UK | 2012 | PubMLST | 11 | 11 | 7-2 | 14 | F1-1 | Cluster 2 | 387 | 244 | 13 | 5 | 17 | 22 | N.D. | N.D. |
| **28142** | UK | 2013 | PubMLST | 11 | 11 | 5 | 2 | F1-146 | Cluster 2 | 1 | 244 | 488 | 5 | 17 | 22 | N.D. | N.D. |
| **28159** | UK | 2013 | PubMLST | 11 | 11 | 21 | 16 | F1-1 | Cluster 2 | 12 | 455 | 13 | 5 | 17 | 22 | N.D. | N.D. |
| **28162** | UK | 2013 | PubMLST | 11 | 11 | 5 | 2 | F1-1 | Cluster 2 | 1 | 244 | 13 | 5 | 17 | 1 | N.D. | N.D. |
| **29737** | UK | 2013 | PubMLST | 11 | 11 | 5 | 2 | F1-1 | Cluster 2 | 1 | 244 | 13 | 5 | 17 | 22 | N.D. | N.D. |
| **28134** | UK | 2013 | PubMLST | 11 | 11 | 5 | 2 | F1-1 | Cluster 2 | 1 | 244 | 13 | 5 | 17 | 22 | N.D. | N.D. |
| **28135** | UK | 2013 | PubMLST | 11 | 11 | 5 | 2 | F1-1 | Cluster 2 | 1 | 244 | 13 | 5 | 17 | 22 | N.D. | N.D. |
| **28136** | UK | 2013 | PubMLST | 11 | 11 | 5 | 2 | F1-1 | Cluster 2 | 1 | 244 | 13 | 5 | 17 | 22 | N.D. | N.D. |
| **28138** | UK | 2013 | PubMLST | 11 | 11 | 5 | 2 | F1-1 | Cluster 2 | 1 | 244 | 13 | 5 | 17 | 22 | N.D. | N.D. |
| **28139** | UK | 2013 | PubMLST | 10651 | 11 | 5 | 2 | F1-1 | Cluster 2 | 1 | 244 | 13 | 5 | 17 | 22 | N.D. | N.D. |
| **28141** | UK | 2013 | PubMLST | 11 | 11 | 5 | 2 | F1-1 | Cluster 2 | 1 | 244 | 13 | 5 | 17 | 22 | N.D. | N.D. |
| **28143** | UK | 2013 | PubMLST | 11 | 11 | 5 | 2 | F1-1 | Cluster 2 | 1 | 244 | 13 | 5 | 17 | 22 | N.D. | N.D. |
| **28144** | UK | 2013 | PubMLST | 11 | 11 | 5 | 2 | F1-1 | Cluster 2 | 1 | 244 | 13 | 5 | 17 | 22 | N.D. | N.D. |
| **28146** | UK | 2013 | PubMLST | 10651 | 11 | 5 | 2 | F1-1 | Cluster 2 | 1 | 244 | 13 | 5 | 17 | 22 | N.D. | N.D. |
| **28147** | UK | 2013 | PubMLST | 11 | 11 | 5 | 2 | F1-1 | Cluster 2 | 1 | 244 | 13 | 5 | 17 | 22 | N.D. | N.D. |
| **28148** | UK | 2013 | PubMLST | 10651 | 11 | 5 | 2 | F1-1 | Cluster 2 | 1 | 244 | 13 | 5 | 17 | 22 | N.D. | N.D. |
| **28149** | UK | 2013 | PubMLST | 11 | 11 | 5 | 2 | F1-1 | Cluster 2 | 1 | 244 | 13 | 5 | 17 | 22 | N.D. | N.D. |
| **28150** | UK | 2013 | PubMLST | 11 | 11 | 5 | 2 | F1-1 | Cluster 2 | 1 | 244 | 13 | 5 | 17 | 22 | N.D. | N.D. |
| **28151** | UK | 2013 | PubMLST | 11 | 11 | 5 | 2 | F1-1 | Cluster 2 | 1 | 244 | 13 | 5 | 17 | 22 | N.D. | N.D. |
| **28152** | UK | 2013 | PubMLST | 11 | 11 | 5 | 2 | F1-1 | Cluster 2 | 1 | 244 | 13 | 5 | 17 | 22 | N.D. | N.D. |
| **28153** | UK | 2013 | PubMLST | 11 | 11 | 5 | 2 | F1-1 | Cluster 2 | 1 | 244 | 13 | 5 | 17 | 22 | N.D. | N.D. |
| **28154** | UK | 2013 | PubMLST | 11 | 11 | 5 | 2 | F1-1 | Cluster 2 | 1 | 244 | 13 | 5 | 17 | 22 | N.D. | N.D. |
| **28156** | UK | 2013 | PubMLST | 11 | 11 | 5 | 2 | F1-1 | Cluster 2 | 1 | 244 | 13 | 5 | 17 | 22 | N.D. | N.D. |
| **28161** | UK | 2013 | PubMLST | 11 | 11 | 5 | 2 | F1-1 | Cluster 2 | 1 | 244 | 13 | 5 | 17 | 22 | N.D. | N.D. |
| **28164** | UK | 2013 | PubMLST | 11 | 11 | 5 | 2 | F1-1 | Cluster 2 | 1 | 244 | 13 | 5 | 17 | 22 | N.D. | N.D. |
| **29721** | UK | 2013 | PubMLST | 11 | 11 | 5 | 2 | F1-1 | Cluster 2 | 1 | 244 | 13 | 5 | 17 | 22 | N.D. | N.D. |
| **29722** | UK | 2013 | PubMLST | 11 | 11 | 5 | 2 | F1-1 | Cluster 2 | 1 | 244 | 13 | 5 | 17 | 22 | N.D. | N.D. |
| **29724** | UK | 2013 | PubMLST | 11 | 11 | 5 | 2 | F1-1 | Cluster 2 | 1 | 244 | 13 | 5 | 17 | 22 | N.D. | N.D. |
| **29726** | UK | 2013 | PubMLST | 10651 | 11 | 5 | 2 | F1-1 | Cluster 2 | 1 | 244 | 13 | 5 | 17 | 22 | N.D. | N.D. |
| **29727** | UK | 2013 | PubMLST | 11 | 11 | 5 | 2 | F1-1 | Cluster 2 | 1 | 244 | 13 | 5 | 17 | 22 | N.D. | N.D. |
| **29728** | UK | 2013 | PubMLST | 11 | 11 | 5 | 2 | F1-1 | Cluster 2 | 1 | 244 | 13 | 5 | 17 | 22 | N.D. | N.D. |
| **29731** | UK | 2013 | PubMLST | 11 | 11 | 5 | 2 | F1-1 | Cluster 2 | 1 | 244 | 13 | 5 | 17 | 22 | N.D. | N.D. |
| **29732** | UK | 2013 | PubMLST | 11 | 11 | 5 | 2 | F1-1 | Cluster 2 | 1 | 244 | 13 | 5 | 17 | 22 | N.D. | N.D. |
| **29733** | UK | 2013 | PubMLST | 11 | 11 | 5 | 2 | F1-1 | Cluster 2 | 1 | 244 | 13 | 5 | 17 | 22 | N.D. | N.D. |
| **29734** | UK | 2013 | PubMLST | 11 | 11 | 5 | 2 | F1-1 | Cluster 2 | 1 | 244 | 13 | 5 | 17 | 22 | N.D. | N.D. |
| **29735** | UK | 2013 | PubMLST | 11 | 11 | 5 | 2 | F1-1 | Cluster 2 | 1 | 244 | 13 | 5 | 17 | 22 | N.D. | N.D. |
| **29736** | UK | 2013 | PubMLST | 11 | 11 | 5 | 2 | F1-1 | Cluster 2 | 1 | 244 | 13 | 5 | 17 | 22 | N.D. | N.D. |

^1^ST: Sequence type, as determined by multi-locus sequence typing.

^2^CC: Clonal complex.

^3^16S: 16S ribosomal DNA type.

^4^PorA VR: PorA variable region.

^5^Phylogenetic cluster- based on phylogenetic and antigen gene similarity to the Hajj reference strain *M7124* (see main text for details)*.*

^6^16S – 16S ribosomal RNA gene typing

^7^SNPs: number of single nucleotide polymorphisms, relative to the Hajj reference strain, *M7124*.

^8^*Neisseria* Base: A database of *Neisseria* genomes (http://nbase.biology.gatech.edu/).

^9^PubMLST: Neisseria Multi Locus Sequence Typing website (<http://pubmlst.org/neisseria>).

^10^U. A. – Allele number not assigned.

^11^N.D. – not done

*Allele numbers were obtained from [*www.pubmlst.org*](http://www.pubmlst.org)*/neisseria*

**Supplementary Table 3: List of six Cluster 1 isolates that shared only three out of four recombinant regions with *M7124*.** Percent identity, alignment length, mismatches and gaps were obtained using BLASTn.

| **Isolate ID** | **Country** | **Year** | **Recombinant region*** | **Percent identity** | **Alignment length** | **Mismatches** | **Gaps** |
| --- | --- | --- | --- | --- | --- | --- | --- |
| 21583 | South Africa | 2011 | 1 | 82.98 | 1163 | 178 | 5 |
| 29326 | South Africa | 2005 | 1 | 82.98 | 1163 | 178 | 5 |
| 29336 | South Africa | 2010 | 1 | 82.98 | 1163 | 178 | 5 |
| 29402 | South Africa | 2013 | 1 | 82.98 | 1163 | 178 | 5 |
| 29387 | South Africa | 2013 | 3 | 99.1 | 4016 | 34 | 1 |
| 29393 | South Africa | 2008 | 4 | 98.23 | 2031 | 36 | 0 |

*Recombinant region numbers correspond to those on Table 1

**Supplementary Table 4:** List of 154 meningococcal isolates included in SNP based maximum likelihood phylogenetic tree shown in Supplementary Figure 2. Table shows location and year of isolation, serogroup and multilocus sequence type (MLST) for each isolate. Empty cells indicate missing information.

| **Isolate ID** | **Location** | **Year** | **Sequence Type (MLST)** | **Clonal complex (MLST)** | **Serogroup** |
| --- | --- | --- | --- | --- | --- |
| 87255 | Tunisia | 1987 | 10308 | 11 | B |
| 98080 | Saudi Arabia | 1998 | 658 | 11 | B |
| NM0029 | USA | 1992 | 11 | 11 | C |
| NM23 | USA | 1992 | 11 | 11 | C |
| NM32 | USA | 1993 | 11 | 11 | C |
| NM35 | USA | 1993 | 11 | 11 | C |
| NM36 | USA | 1993 | 11 | 11 | C |
| NM43 | USA | 1993 | 11 | 11 | C |
| NM0084 | USA | 1995 | 11 | 11 | C |
| NM82 | USA | 1995 | 11 | 11 | C |
| NM94 | USA | 1995 | 11 | 11 | C |
| NM95 | USA | 1995 | 11 | 11 | C |
| NM126 | USA | 1996 | 11 | 11 | C |
| NM133 | USA | 1996 | 11 | 11 | C |
| NM313 | USA | 2003 | 11 | 11 | C |
| NM1482 | USA | 2003 | 1287 | 11 | C |
| NM1495 | USA | 2003 | 11 | 11 | C |
| NM586 | USA |  | 11 | 11 | C |
| NM762 | USA |  | 11 | 11 | C |
| NM3682 | UK | 1970 | 11 | 11 | W |
| NM3684 | Netherlands | 1985 | 1287 | 11 | W |
| NM3680 | Mali | 1994 | 11 | 11 | W |
| NM3681 | Gambia | 1995 | 11 | 11 | W |
| NM3685 | Indonesia | 1996 | 11 | 11 | W |
| NM3687 | Brazil | 1997 | 11 | 11 | W |
| NM174 | USA | 1998 | 11 | 11 | W |
| 2001001 | Algeria | 1999 | 11 | 11 | W |
| M7124 | Saudi Arabia | 2000 | 11 | 11 | W |
| M7089 | USA | 2000 | 11 | 11 | W |
| 2000081 | Senegal | 2000 | 11 | 11 | W |
| 2000175 | Cameroon | 2000 | 11 | 11 | W |
| 2001072 | Mauritius | 2001 | 11 | 11 | W |
| 2001073 | Mauritius | 2001 | 11 | 11 | W |
| 2001213 | Burkina Faso | 2001 | 11 | 11 | W |
| 2001068 | Chad | 2001 | 11 | 11 | W |
| NM3686 | Brazil | 2001 | 11 | 11 | W |
| M9261 | Burkina Faso | 2001 | 11 | 11 | W |
| NM3688 | Brazil | 2001 | 11 | 11 | W |
| 2002004 | France | 2002 | 11 | 11 | W |
| 2004032 | Benin | 2004 | 11 | 11 | W |
| 2004264 | Burkina Faso | 2004 | 11 | 11 | W |
| 2005079 | Chad | 2005 | 11 | 11 | W |
| 2005040 | Chad | 2005 | 11 | 11 | W |
| NM3147 | USA | 2009 | 11 | 11 | W |
| M12611 | Chile | 2011 | 11 | 11 | W |
| M1412 | Chile | 2012 | 11 | 11 | W |
| NM27 | USA | 1993 | 1622 | 23 | Y |
| NM51 | USA | 1993 | 23 | 23 | Y |
| NM90 | USA | 1995 | 23 | 23 | Y |
| NM80 | USA | 1995 | 1621 | 23 | Y |
| NM115 | USA | 1996 | 23 | 23 | Y |
| NM165 | USA | 1997 | 23 | 23 | Y |
| NM271 | USA | 2001 | 23 | 23 | Y |
| NM3042 | USA | 2007 | 23 | 23 | Y |
| NM3131 | USA | 2008 | 893 | 23 | Y |
| NM3222 | USA | 2009 | 23 | 23 | Y |
| NM3144 | USA | 2009 | 6799 | 23 | Y |
| NM3158 | USA | 2009 | 6800 | 23 | Y |
| NM3164 | USA | 2009 | 3582 | 23 | Y |
| NM3223 | USA | 2009 | 23 | 23 | Y |
| 9757 | Brazil | 1988 | 33 | 32 | B |
| 4119 | Brazil | 1989 | 639 | 32 | B |
| NM1476 | USA | 2000 | 32 | 32 | B |
| NM477 | USA | 2001 | 32 | 32 | B |
| 2002030 | Cameroon | 2002 | 32 | 32 | B |
| NM418 | USA | 2003 | 32 | 32 | B |
| NM422 | Oregon, USA | 2003 | 32 | 32 | B |
| NM3141 | USA | 2009 | 1249 | 32 | B |
| NM3173 | USA | 2009 | 32 | 32 | B |
| 2002020 | Cameroon | 2001 | 32 | 32 | C |
| 12888 | Brazil | 2004 | 639 | 32 | C |
| 9506 | Brazil | 2004 | 33 | 32 | C |
| M13265 | USA | 2004 | 32 | 32 | C |
| M13255 | USA | 2005 | 32 | 32 | C |
| NM003 | USA | 1992 | 437 | 44 | B |
| NM2657 | USA | 2007 | 60 | 60 | Not groupable |
| NM140 | USA | 1996 | 2981 | 103 | Z |
| NM183 | USA | 1998 | 2982 | 103 | Z |
| NM2781 | USA | 1998 | 6937 | 103 | Z |
| NM576 | USA | 1999 | 5467 | 103 | Z |
| 2003051 | Niger | 2003 | 2880 | 167 | Y |
| NM134 | USA | 1996 | 185 | 174 | W |
| 2007461 | Togo | 2007 | 2881 | 175 | W |
| NM255 | USA | 2000 | 2980 | 175 | X |
| 80179 | France | 1980 | 178 | 178 | E |
| 97014 | Niger | 1997 | 181 | 181 | X |
| 98002 | Niger | 1998 | 181 | 181 | X |
| 2002038 | Niger | 2002 | 181 | 181 | X |
| 2005172 | Niger | 2005 | 181 | 181 | X |
| 2006087 | Niger | 2006 | 5789 | 181 | X |
| 2008223 | Burkina Faso | 2007 | 181 | 181 | X |
| NM2795 | USA | 1998 | 198 | 198 | Not groupable |
| 98008 | France | 1998 | 461 | 461 | E |
| NM3001 | USA | 2006 | 1157 | 1157 | Not groupable |
| NM045 | USA | 1993 | 136 | 41/44 | B |
| 96037 | Cameroon | 1996 | 291 | 41/44 | B |
| NM151 | USA | 1997 | 41 | 41/44 | B |
| NM2866 | USA | 1998 | 6939 | 41/44 | B |
| NM518 | USA | 2002 | 44 | 41/44 | B |
| NM0552 | USA | 2003 | 170 | 41/44 | B |
| NM3139 | USA | 2009 | 4682 | 41/44 | B |
| NM3230 | USA | 2009 | 154 | 41/44 | B |
| 77221 | Burkina Faso | 1997 | 8798 | Not defined | A |
| 73696 | Brazil | 1973 | 8813 | Not defined | C |
| 81858 | Vietnam | 1981 | 344 | Not defined | C |
| NM3081 | USA | 2007 | 6173 | Not defined | Not groupable |
| 92045 | France | 1992 | 4122 | Not defined |  |
| 93003 | France | 1993 | 4959 | Not defined |  |
| 93004 | France | 1993 | 5594 | Not defined |  |
| 61103 | Niger | 1961 | 57 | 1 | A |
| 61106 | Niger | 1961 | 1 | 1 | A |
| 63023 | Niger | 1963 | 57 | 1 | A |
| 69096 | Mali | 1969 | 1 | 1 | A |
| 69155 | Algeria | 1969 | 8973 | 1 | A |
| 69176 | Algeria | 1969 | 8973 | 1 | A |
| 69100 | Mali | 1969 | 1 | 1 | A |
| 70012 | Chad | 1970 | 1 | 1 | A |
| 70021 | Algeria | 1970 | 1 | 1 | A |
| 70082 | Niger | 1970 | 1 | 1 | A |
| 70030 | Burkina Faso | 1992 | 1 | 1 | A |
| 96060 | Algeria | 1992 | 1 | 1 | A |
| 68094 | New Zealand | 1968 | 1 | 1 | B |
| 69166 | USA | 1969 | 1 | 1 | Z |
| 63049 | USA | 1915 | 4 | 4 | A |
| 63006 | Burkina Faso | 1963 | 4 | 4 | A |
| 63041 | Chad | 1963 | 4 | 4 | A |
| 64182 | Niger | 1964 | 4 | 4 | A |
| 65014 | Niger | 1965 | 4 | 4 | A |
| 65012 | Niger | 1965 | 4 | 4 | A |
| 97027 | Niger | 1972 | 4 | 4 | A |
| 75643 | Brazil | 1975 | 5 | 5 | A |
| 75689 | Brazil | 1975 | 5 | 5 | A |
| 88050 | Chad | 1988 | 5 | 5 | A |
| 94018 | Congo | 1994 | 5 | 5 | A |
| 96023 | Niger | 1996 | 5 | 5 | A |
| 96024 | Niger | 1996 | 5 | 5 | A |
| 97020 | Mali | 1997 | 5 | 5 | A |
| 97021 | Chad | 1997 | 7 | 5 | A |
| 97018 | Mali | 1997 | 5 | 5 | A |
| 97008 | Niger | 1997 | 5 | 5 | A |
| 98005 | Niger | 1998 | 5 | 5 | A |
| 2000080 | Algeria | 2000 | 7 | 5 | A |
| 2000063 | Niger | 2000 | 7 | 5 | A |
| NM604 | USA | 2000 | 7 | 5 | A |
| 2001212 | Burkina Faso | 2001 | 7 | 5 | A |
| NM606 | USA | 2001 | 7 | 5 | A |
| NM607 | USA | 2001 | 7 | 5 | A |
| 2002007 | Niger | 2002 | 7 | 5 | A |
| NM3642 | Bangladesh | 2003 | 4789 | 5 | A |
| 2003022 | Niger | 2003 | 7 | 5 | A |
| 2004090 | Niger | 2004 | 7 | 5 | A |
| 2004085 | Niger | 2004 | 7 | 5 | A |
| NM3652 | Bangladesh | 2006 | 8428 | 5 | A |
| 2007056 | Burkina Faso | 2007 | 2859 | 5 | A |
